# Identification of Trans-Sialidases as a Common Mediator of Endothelial Cell Activation by African Trypanosomes

**DOI:** 10.1371/journal.ppat.1003710

**Published:** 2013-10-10

**Authors:** Zeinab Ammar, Nicolas Plazolles, Théo Baltz, Virginie Coustou

**Affiliations:** French National Centre for Scientific Research (CNRS), Université Bordeaux Segalen, Microbiologie fondamentale et Pathogénicité, UMR 5234, Bordeaux, France; Washington University School of Medicine, United States of America

## Abstract

Understanding African Trypanosomiasis (AT) host-pathogen interaction is the key to an “anti-disease vaccine”, a novel strategy to control AT. Here we provide a better insight into this poorly described interaction by characterizing the activation of a panel of endothelial cells by bloodstream forms of four African trypanosome species, known to interact with host endothelium. *T. congolense*, *T. vivax*, and *T. b. gambiense* activated the endothelial NF-κB pathway, but interestingly, not *T. b. brucei*. The parasitic TS (trans-sialidases) mediated this NF-κB activation, remarkably *via* their lectin-like domain and induced production of pro-inflammatory molecules not only *in vitro* but also *in vivo*, suggesting a considerable impact on pathogenesis. For the first time, TS activity was identified in *T. b. gambiense* BSF which distinguishes it from the subspecies *T. b. brucei.* The corresponding TS were characterized and shown to activate endothelial cells, suggesting that TS represent a common mediator of endothelium activation among trypanosome species with divergent physiopathologies.

## Author Summary

African trypanosomiasis remains by far the most devastating parasitic disease in Africa affecting both humans and livestock. The current control strategies are not efficient because of the increasing resistance to trypanocidal drugs, and the antigenic variation that impedes vaccine development. An alternative strategy aiming to neutralize the pathological effects of the parasite rather than eliminate it was proposed. Therefore, it is essential to understand the development of pathogenesis and characterize the pathogenic factors. In this context, we wanted to elucidate the host-pathogen interaction between the African trypanosomes and the mammalian host endothelium. For the first time, we clearly demonstrated that animal African trypanosomes activate the endothelial cells via the NF-κB pathway and cause a pro-inflammatory response *in vitro* and *in vivo* via their TS. By comparing four different trypanosomes species, we showed that they displayed distinct capacities for activation. For the first time, we identified sialidase activity in the human parasite *T. brucei gambiense* and showed that sialidases are the mediators of this endothelial activation, in both human and animal trypanosomes. Interestingly, the lectin-like domain of this enzyme was responsible for the activation rather than the catalytic site. This study brings considerable insights into the host-pathogen relationship and designates the sialidases as a perfect target for an anti-disease strategy.

## Introduction

Animal African trypanosomiasis (AAT) is a severe disease affecting livestock in sub-Saharan Africa throughout an area of approximately 10 million km^2^, and causing annual economic losses of several billion dollars [1], [2]. The disease is characterized by severe anaemia, weight loss and immunosupression, leading to the death of the animal if not treated. It is caused by the parasites *Trypanosoma congolense*, *Trypanosoma vivax* and to a lesser extent, *Trypanosoma brucei*. Human African trypanosomiasis (HAT), also known as sleeping sickness, affects mostly poor populations living in rural areas of Africa with ∼10 000 new cases reported per year according to World Health Organization reports. Patients suffer from progressive neurological dysfunction that culminates in death. Almost 90% of the reported HAT cases are caused by *Trypanosoma brucei gambiense*.

During the course of infection, African trypanosomes remain exclusively extracellular and are found intermittently in the blood of the mammalian host as bloodstream forms (BSF). Therefore, these parasites are necessarily in contact, either directly or indirectly, with the endothelial cells. At a certain stage of infection, *T. b. gambiense* and *T. vivax* invade internal organs, including the central nervous system, which requires direct contact with the endothelial cells of blood brain barrier (BBB) [3], [4]. On the contrary, *T. congolense* remains exclusively intravascular, but binds to the walls of capillaries of infected cattle and to bovine aortic endothelial cells (BAE) *in vitro* [5], [6].

The vascular endothelium is not only a permeability barrier but also a multifunctional “organ” that, among other functions, plays a critical role in regulating the immune response [7]. Endothelial cell activation is a central pathophysiological process allowing the endothelium to participate in an inflammatory response by triggering activation pathways of the cell leading to a rapid up-regulation of gene transcription. Furthermore, endothelial cells from different locations present a phenotypic variation and can generate different responses to the same stimulus.

NF-κB is an essential and well characterized family of transcription factors, conserved from human to *Drosophila*, and known to be a central mediator for numerous cell functions including the immune response [8], [9], [10]. Under normal conditions, NF-κB is bound to IκB (NF-κB inhibitor) and retained in the cytoplasm. In an infection context, a cascade of kinases is activated by the pathogen, leading to IκB phosphorylation and NF-κB translocation to the nucleus, where it induces the expression of NF-κB-responsive genes. Most products of these genes are crucial for the immune system and specifically for the establishment of an inflammatory response, such as pro-inflammatory cytokines, nitric oxide synthase and leukocyte adhesion molecules. The NF-κB pathway plays a prominent role in regulating the immune response to parasite infections [11]. NF-κB-dependent response of endothelial cells has been described for *Trypanosoma cruzi*, contributing to cell invasion and inflammation [12], [13], which are important components in the development of congestive heart failure observed during Chagas' disease [14]. Likewise, the host's inflammatory response is a major pathophysiological factor in disease progression during African trypanosomiasis, hence the outcomes of this activation on the host pathogen relationship could be considerable. However, to our knowledge, no studies have yet examined the activation of endothelium by African trypanosomes, with the exception of one study on endothelial activation by *T. b. gambiense* [15], which was limited to a single endothelial cell model with no detailed characterization and did not further investigate the molecular mediators of activation. Such studies are of major interest as they contribute to the understanding of the host-pathogen relationship, which is the key to the “anti-disease strategy” to fight African trypanosomiasis based on neutralizing the pathological effects rather than eliminating the parasites. This concept was proposed as an alternative to control trypanosomiasis after the failure of classical chemotherapy and vaccine [16].

Sialidases (SA) and trans-sialidases (TS) are proposed as pathogenic factors in AAT [17] and their involvement in virulence has been extensively described in *T. cruzi* [18]. In African trypanosomes, their role has been well established in the insect stages, where they mediate a trans-sialylation process (transfer of carbohydrate-linked sialic acids to an acceptor sugar on the parasite surface) of their major glycoproteins to form a protective coat that is essential for parasite survival in the fly gut [19], [20]. However, expression of TS and SA active enzymes was not clearly demonstrated in BSF of *T. vivax* and *T. congolense* and their role in mammalian hosts was not elucidated until recently [21], [22]. In fact, SA/TS activities result from active secretion with a correlation with parasite load in the blood but also from passive release after immune-mediated lysis of the parasite, and fluctuate throughout the course of infection in the mammalian hosts. During this stage, SA and TS play a crucial role in the infection process, most critically in anaemia, via erythrocyte desialylation [22], [23], [24]. Moreover, TS were shown to induce endothelial cell and B lymphocyte activation by *T. cruzi* [13], [25], and given its prominent role as virulence factor in AAT, the newly identified TS of African trypanosomes BSF seemed to be appropriate candidates to mediate endothelial cell activation. However, the literature agrees on the absence of TS and SA activities in BSF of *T. b. brucei*, and to our knowledge no studies regarding SA and TS activities have been performed on *T. b. gambiense* BSF.

Here we compared the capacity for endothelial activation of four different species of African trypanosomes, using primary cultures of BAE and both human and murine endothelial cell lines. These African trypanosome species, known to cause different physiopathologies, had distinct activation capacities, interestingly in correlation with the presence of SA/TS activity. Specifically, *T. b. brucei* BSF did not activate the endothelial cells, whereas *T. vivax*, *T. congolense* and *T. b. gambiense* BSF were capable of endothelial cell activation *via* the NF-κB pathway. The different endothelial cell models we used allowed identification of organ and species specificities while characterizing the endothelial cell activation process. Kinetic patterns of activation and clear quantification of activated cells were established for the first time regarding an African trypanosomes/endothelial cell interaction study. We clearly demonstrated the presence of enzymatic TS and SA activity in *T. b. gambiense* BSF and showed involvement of TS, most likely through their lectin-like domain, in endothelial cell activation, and subsequently in inflammation. Most importantly, our findings pointed towards the TS as a common mediator of activation among different species of African trypanosomes. These data reinforced the role of these enzymes as virulence factors involved, not only in anaemia, *via* their catalytic properties but also in inflammation development *via* their lectin domain. Moreover, these findings render TS a potential vaccine target, and given the established role of TS in the interaction of *T. cruzi* with its host [26], [27], this vaccine could ideally serve against both American and African trypanosomes. Lastly, this study offered new insights into the host-pathogen interaction in African trypanosomiasis, which is essential for any attempt to control this disease.

## Results

### Endothelial cells are activated by *T. congolense*, *T. vivax*, *T. b. gambiense* but not *T. b. brucei*

**Figure 1:**
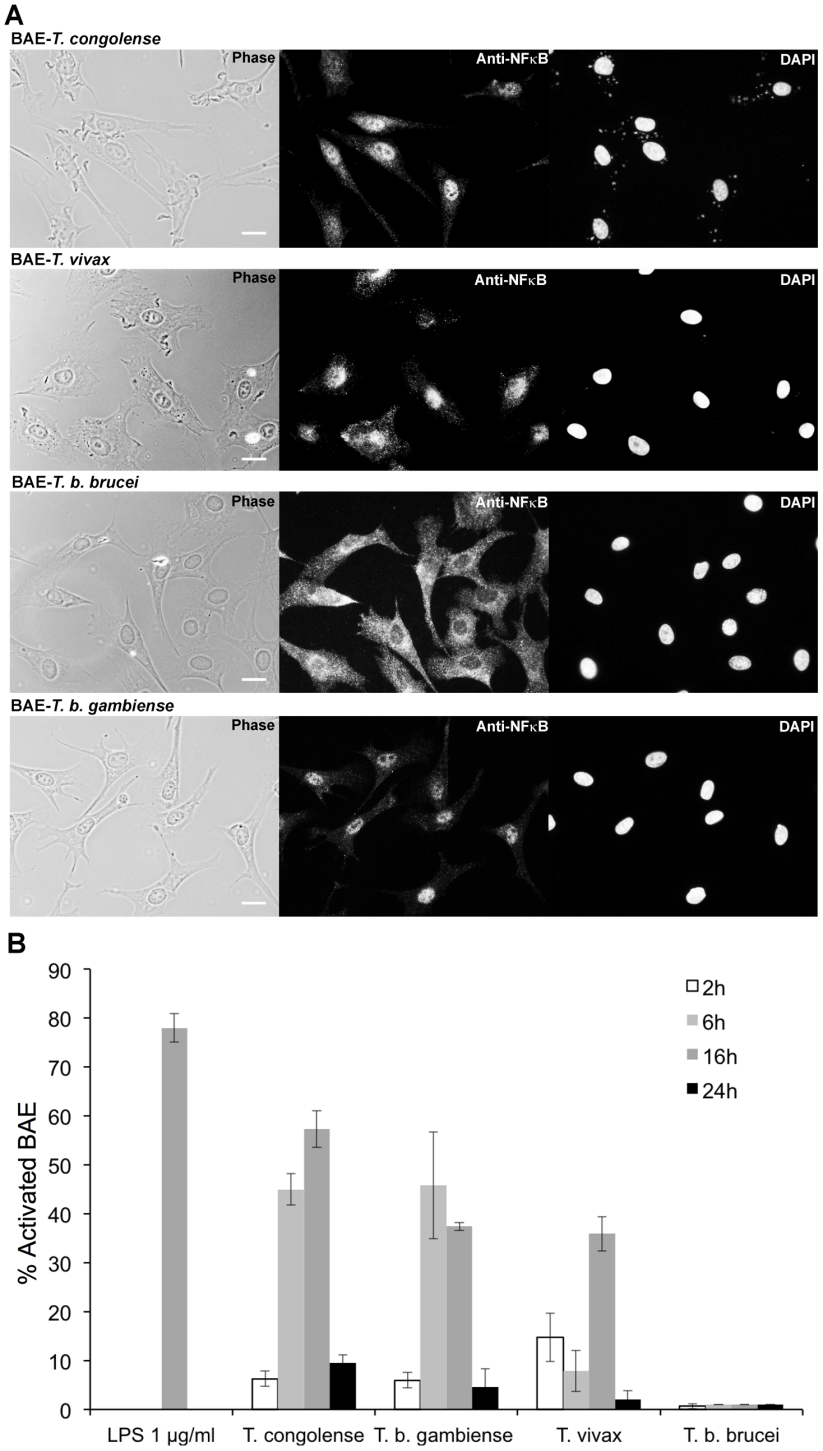
Activation of BAE by African trypanosomes. (A) NF-κB immunofluorescent staining on BAE after 16 h of coculture with *T. congolense* IL3000, *T. vivax* Y486, *T. b. brucei* AnTat 1.1 and *T. b. gambiense* 1135 BSF. Scale bars = 20 µM. (B) Kinetics of BAE activation: percentage of activated BAE in presence of 1 µg/ml LPS and after 2, 6, 16 and 24 h of coculture with BSF of *T. congolense* IL3000, *T. vivax* Y486, *T. b. brucei* AnTat 1.1 and *T. b. gambiense* 1135. Results were similar with *T. congolense* STIB910 strain, *T. b. gambiense* LiTat strain, and *T. b. brucei* 427 strain. Each experiment was repeated at least three times independently. Results are expressed as mean-values±standard deviation (SD).

To determine whether African trypanosomes can activate endothelial cells, primary cultures of BAE were first cocultivated with four species of African trypanosomes: BSF of *T. congolense* (two isolates IL3000 and STIB910), *T. vivax* (Y486), *T. b. gambiense* (1135 and LiTat) and *T. b. brucei* (AnTat 1.1 and 427). BAE were previously cultivated for 24 h in culture medium without serum to avoid the interference of serum components with activation of NF-κB. NF-κB translocation to the nucleus was studied by indirect immunofluorescence as an indicator of cell activation. For BAE in culture medium alone (control cells), NF-κB staining was mainly limited to the cytoplasm (data not shown). In the presence of *T. congolense*, *T. vivax* and *T. b. gambiense*, the nucleus of the majority of BAE became stained (Fig. 1A). Interestingly, staining was limited to the cytoplasm in the presence of *T. b. brucei*.

The percentage of activated cells (with stained nuclei) was determined by counting approximately 500 cells for each condition and at various time points, in order to establish the activation kinetics. Data was then normalized to the control by retrieving the percentage of cells activated in culture medium alone (control) representing the background activation (Fig. 1B). 78±2.96% of BAE were activated in the presence of LPS (positive control). Activation by both *T. congolense* and *T. b. gambiense* started after 6 h of coculture, with 45±3.2% and 48.5±10.9% of cells activated respectively, whereas *T. vivax* activated BAE only after 16 h of coculture, with 35.9±3.5% of BAE activated. However, the number of parasites used in *T. vivax* activation assays was 10 times lower than for the other species, due to experimental limitations, which could explain the delay observed in activation kinetics. Finally, *T. b. brucei* did not activate BAE at all. Note that after 24 h of coculture, an increased rate of activation by serum components was observed in control cells and used to normalize the quantification, therefore results at this particular time point are probably underestimated.

### Heterogeneity of endothelial cells reflects on NF-κB activation by African trypanosomes

**Figure 2:**
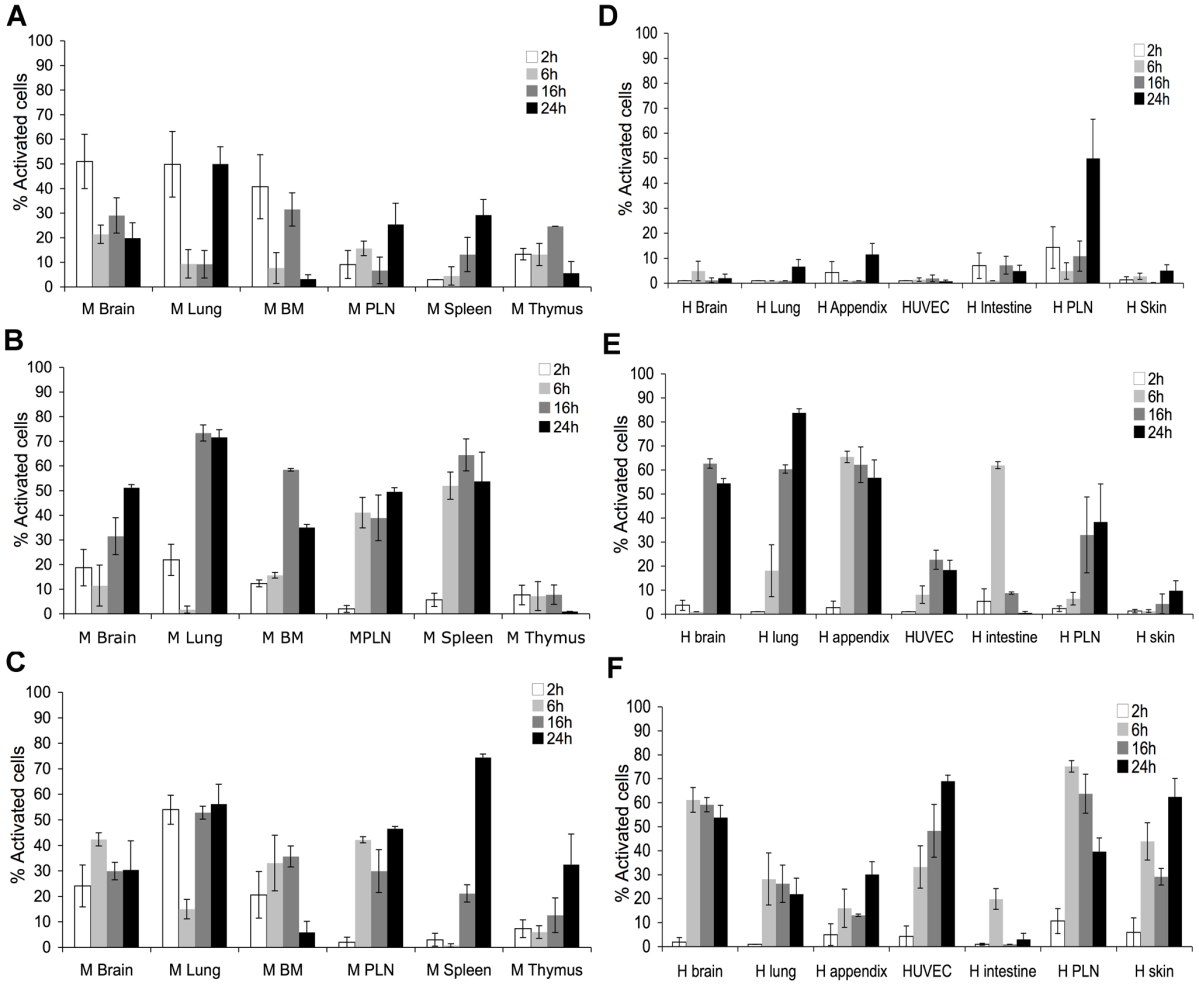
Kinetics of activation of murine and human endothelial cells by *T. congolense*, *T. b. gambiense* and *T. vivax* BSF. Murine (M) (A, B, and C) and human (H) (D, E, and F) endothelial cells from brain, lung, BM (bone marrow), spleen, PLN (peripheral lymph node), thymus, appendix, umbilical vein (HUVEC), intestine and skin were cultivated for 2, 6, 16 and 24 h with BSF *T. congolense* IL3000 (A and D), *T. vivax* Y486 (B and E) and *T. b. gambiense* 1135 (C and F). Results were similar with *T. congolense* STIB910 strain, *T. b. gambiense* LiTat strain, and *T. b. brucei* 427 strain. Percentage of activated endothelial cells at each time point is represented as mean value±SD of at least three independent experiments. See also Fig. S1 and S2, and Table S1.

Since heterogeneity of endothelial cells from different organs and species is well known [28], [29], [30], it was imperative to analyse a large panel of endothelial cells. Here, a primary culture of BAE was studied as a preferred model, since cattle are a natural host for animal African trypanosomes. In addition, we tested 13 different organ-specific endothelial cell lines, from either human (H) or murine (M) origins: they are microvascular endothelial cells, with the exception of HUVEC and H and M Peripheral Lymph Nodes (PLN). H and M PLN also represent the high endothelial cells (HEC) phenotype. These cells have conserved endothelial cells properties, more specifically morphological characteristics (microscope observation), endothelial cell markers [31], and a functional TNFα-induced NF-κB cell signaling pathway (Fig. S1A and B). Results showed that *T. b. brucei* did not activate the tested endothelial cells, regardless of their origin (Fig. S1C and D), whereas *T. b. gambiense* did (Fig. 2), thus confirming the results seen with the BAE. Interestingly, *T. congolense* activated murine but not human endothelial cells, with the exception of H PLN, which could be explained by its HEC phenotype (Fig. 2 and S2, Table S1). This suggests that activation by *T. congolense* is species-specific. Furthermore, *T. congolense* seemed to activate murine cells with preference for some tissues: M Brain, M Lung and M bone marrow (BM) were the most activated, 51%, 49.8%, and 40% of cells activated respectively, while M PLN, M Spleen and M Thymus were less activated, 25%, 29%, 24.6% of cells activated, respectively. Results with *T. vivax* also showed organ specificity: all endothelial cell lines were activated but neither M Thymus nor H Skin cell lines.

The kinetics of activation showed an additional scale of variability between the 13 endothelial cell lines used (Fig. 2). For example, while 2 h of coculture were sufficient for *T. congolense* to activate M Brain, M Lung and M BM; M PLN and M Spleen required 24 h for activation. Not only did the minimum coculture time for activation vary between these endothelial cells, but also the peak of activation, which occurred at different time points depending on cell origin. For instance, M Lung were most activated after 24 h of coculture with *T. congolense*, while M BM were most activated after 16 h of coculture.

To summarize, our results showed that the African trypanosomes *T. congolense*, *T. vivax* and *T. b. gambiense* did activate endothelial cells and, interestingly, *T. b. brucei* did not. Activation of endothelial cells by African trypanosomes was not uniform: specificity for species and for organs was observed, and a proper activation profile for each endothelial cell line was defined according to the activation kinetics.

### NF-κB activation induces a pro inflammatory response in endothelial cells

In order to determine the consequences of NF-κB activation on the endothelial cell, three different markers, potentially regulated by the NF-κB factor, were studied: i) production of pro-inflammatory cytokines IL-1β and IL-6; ii) modification of the expression pattern of adhesion molecules ICAM-1 and VCAM-1; iii) and nitric oxide production by NO synthase. These assays were performed on M BM and Lung, the two cell lines most activated by *T. congolense* and for which commercial antibodies are extensively developed.

**Figure 3:**
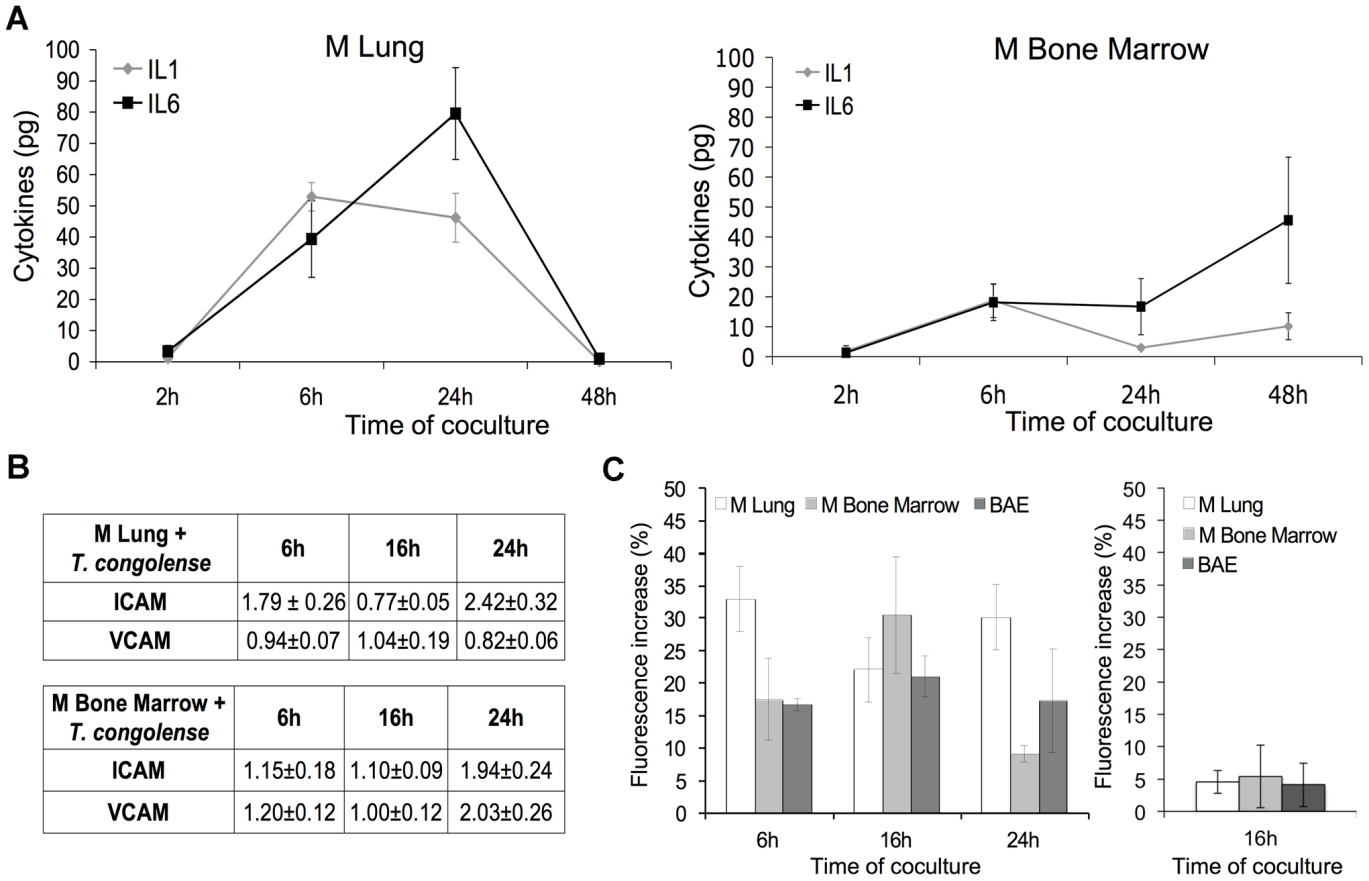
Production of pro-inflammatory molecules as a consequence of NF-κB activation. (A) Secretion of IL-1β and IL-6 by M Lung (left) and M Bone Marrow (BM) (right) microvascular endothelial cells in the supernatant after 2, 6, 24 and 48 h of coculture with BSF of *T. congolense* IL3000. (B) Expression of VCAM-1 and ICAM-1 adhesion molecules on surface of M Lung and M BM microvascular endothelial cells activated by *T. congolense* after 6, 16 and 24 h of coculture. Ratios of MFIs (obtained by flow cytometry analysis) were calculated as detailed in Materials and methods. (C) Production of intracellular nitrite by M Lung, M BM and BAE cocultivated with *T. congolense* in absence (left) or presence of 1 mM Nω-Nitro-L-arginine (right). The basal fluorescence intensity was measured in control wells (unstimulated M Lung, M BM or BAE). Increase of fluorescence intensity in wells containing endothelial cells cocultivated with *T. congolense* is subsequently expressed as percentage relative to control. Data are expressed as mean values ±SD of three independent experiments. See also Fig. S3.

IL-1β and IL-6 produced in the coculture media were measured at different time points (Fig. 3A). As controls, endothelial cell lines were stimulated by 5 ng/ml of TNFα for 4 h (data not shown), or unstimulated to determine basal level of IL-1β and IL-6 (data not shown). In the presence of *T. congolense*, significant amounts of IL 1β were detected after 6 h whereas for IL-6, longer incubation periods were required: 24 h for M Lung and 48 h for M BM. This seemed to reflect the known autocrine effect of IL-1 β on the production of IL-6. M Lung activated with *T. congolense* produced high amounts of IL-1β and IL-6 that reached 52.9±4.56 pg/ml after 6 h of coculture and 102±8.3 pg/ml after 24 h of coculture, respectively. M BM activated with *T. congolense* produced smaller amounts of cytokines: 18.7±6 pg/ml of IL-1β at 6 h and 56.9±26.3 of IL-6 at 48 h of coculture.

The expression of the adhesion molecules ICAM-1 and VCAM-1 on M Lung and M BM was examined by flow cytometry at 6 h, 16 h and 24 h of coculture with *T. congolense* (Fig. 3B). The modification in the expression pattern is evaluated by the ratio of median fluorescence intensity (MFI) of cells in the presence of *T. congolense*, over MFI of unstimulated cells. Results showed that in presence of *T. congolense* ICAM-1 expression in M Lung increased 1.79±0.26 fold at 6 h, and reached 2.42±0.32 fold at 24 h (Fig. 3B). The down regulation of ICAM-1 observed at 16 h is in harmony with the NF-κB activation kinetics of M lung and could be due to ICAM-1 shedding from membrane surface [32]. *T. congolense* had no influence on VCAM-1 expression in M Lung, which is consistent with previous results showing that VCAM-1 is not inducible in this endothelial cell type [29]. ICAM-1 and VCAM-1 expression in M BM increased 1.94±0.24 and 2±0.26 fold, respectively, after 24 h of coculture with *T. congolense*.

Intracellular production of nitric oxide (NO) was measured by fluorometric assay (Fig. 3C). In the presence of *T. congolense*, fluorescence intensity increase reached 32.9±9.2%, 30.4±9% and 20.9±3.1% in M Lung, M BM and BAE respectively. Addition of Nω-Nitro-L-arginine, a specific inhibitor of NO synthase reduced it to 4.5±1.8%, 5.4±4.8% and 4±3.4% respectively (Fig. 3C). This indicates that the observed increase of NO production was a direct result of L-arginine-dependent NO synthase activity.

Lastly, and as expected, *T. b. brucei* that did not activate the endothelial NF-κB pathway was unable to stimulate a pro-inflammatory response by neither M BM nor M Lung as shown by the lack of production of IL-1β and IL-6 and by a nearly absent up-regulation of ICAM-1/VCAM-1 on both endothelial cell types (Fig. S3A and B).

In conclusion, activation of NF-κB induced the production of pro-inflammatory molecules by the endothelial cells, marked by the production of the cytokines IL-1β and IL-6, nitric oxide and expression of adhesion molecules.

### Endothelial cells are activated via the classical NF-κB pathway

To determine which NF-κB pathway (classical or alternative) is responsible for the observed activation and subsequent pro-inflammation, we i) performed all assays regarding the pro-inflammatory response using JSH-23, a specific inhibitor of p65 subunit of NF-κB, ii) examined the phosphorylation of IκB, the inhibitor of NF-κB.

**Figure 4:**
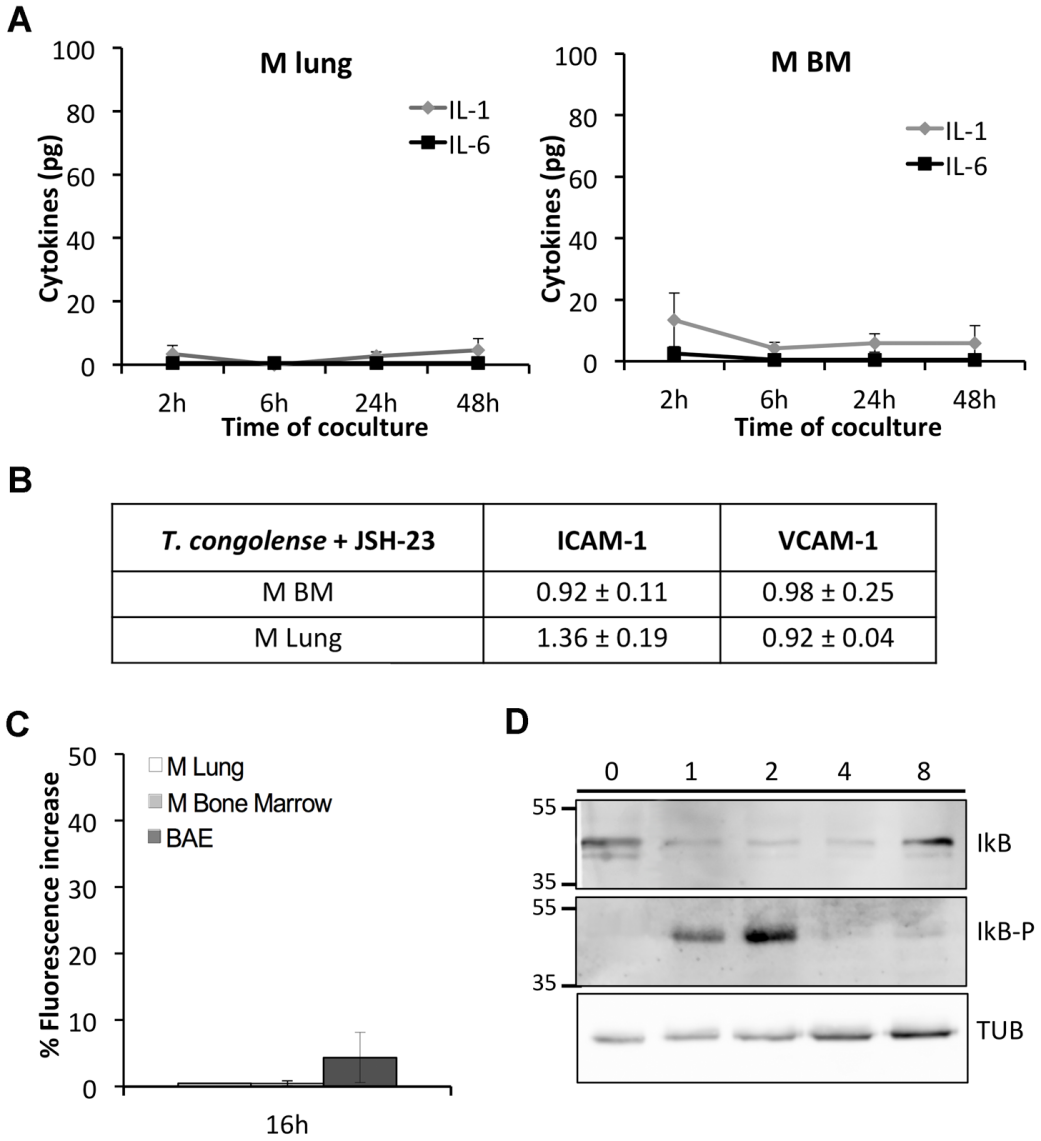
NF-κB activation and its pro-inflammatory consequences are dependent on the classical pathway. Inhibitory effect of JSH-23, a specific inhibitor of the classical NF-κB pathway on (A) secretion of IL-1β and IL-6 by M Lung (left) and M Bone Marrow (BM) (right) microvascular endothelial cells in the supernatant after 2, 6, 24 and 48 h of coculture with BSF of *T. congolense* IL3000 (B) expression of VCAM-1 and ICAM-1 adhesion molecules on surface of M Lung and M BM microvascular endothelial cells after 24 h of coculture with *T. congolense* and (C) production of intracellular nitrite by M Lung, M BM and BAE after 16 h of coculture with *T. congolense*. Data are expressed as mean values ±SD of three independent experiments. (D) Detection of phosphorylated IκBα by immunoblotting. M Lung were cultivated with *T. congolense* for 0, 1, 2, 4 or 8 h, and total protein extracts were subjected to western blotting with anti IκBα (upper panel), anti phospho-IκBα (median panel) or anti-tubulin (lower panel) antibodies.

In presence of JSH-23 the increase of NO, cytokine production, and ICAM-1/VCAM-1 up-regulation were inhibited, indicating that this observed response was a specific consequence of NF-κB activation, and most importantly, *via* the classical pathway. Briefly, in the presence of JSH-23, cytokine secretion by both M BM and M lung cocultivated with *T. congolense* did not exceed 10 pg for IL-1β and 20 pg for IL-6 (Fig. 4A). ICAM-1 and VCAM-1 expression on M BM did not increase after 24 h of coculture with *T. congolense* (Fig. 4B), whereas on M Lung up-regulation of ICAM-1 decreased to 1.36±0.19 fold. In the NO assay, fluorescence intensity increase after 16 h of coculture with *T. congolense* was reduced to 4.4±3.8%, 0.5±0.4% and 0.5% for BAE, M BM and M lung respectively (Fig. 4C).

To explore the molecules involved in upstream NF-κB translocation, we examined the presence of a phosphorylated form of IκB. In M Lung, IκB was not phosphorylated in the presence of *T. b. brucei* (Fig. S3C) whereas in the presence of *T. congolense* a phosphorylated form of IκB is detected after 2 h of coculture (Fig. 4D), in synergy with the NF-κB activation kinetics of M Lung.

Taken together, these data indicate that *T. congolense* activates the endothelial cells via the classical pathway precisely, and that the subsequent pro-inflammatory response is dependent on this specific NF-κB pathway.

### Trypanosome TS are highly involved in endothelial cell activation

**Figure 5:**
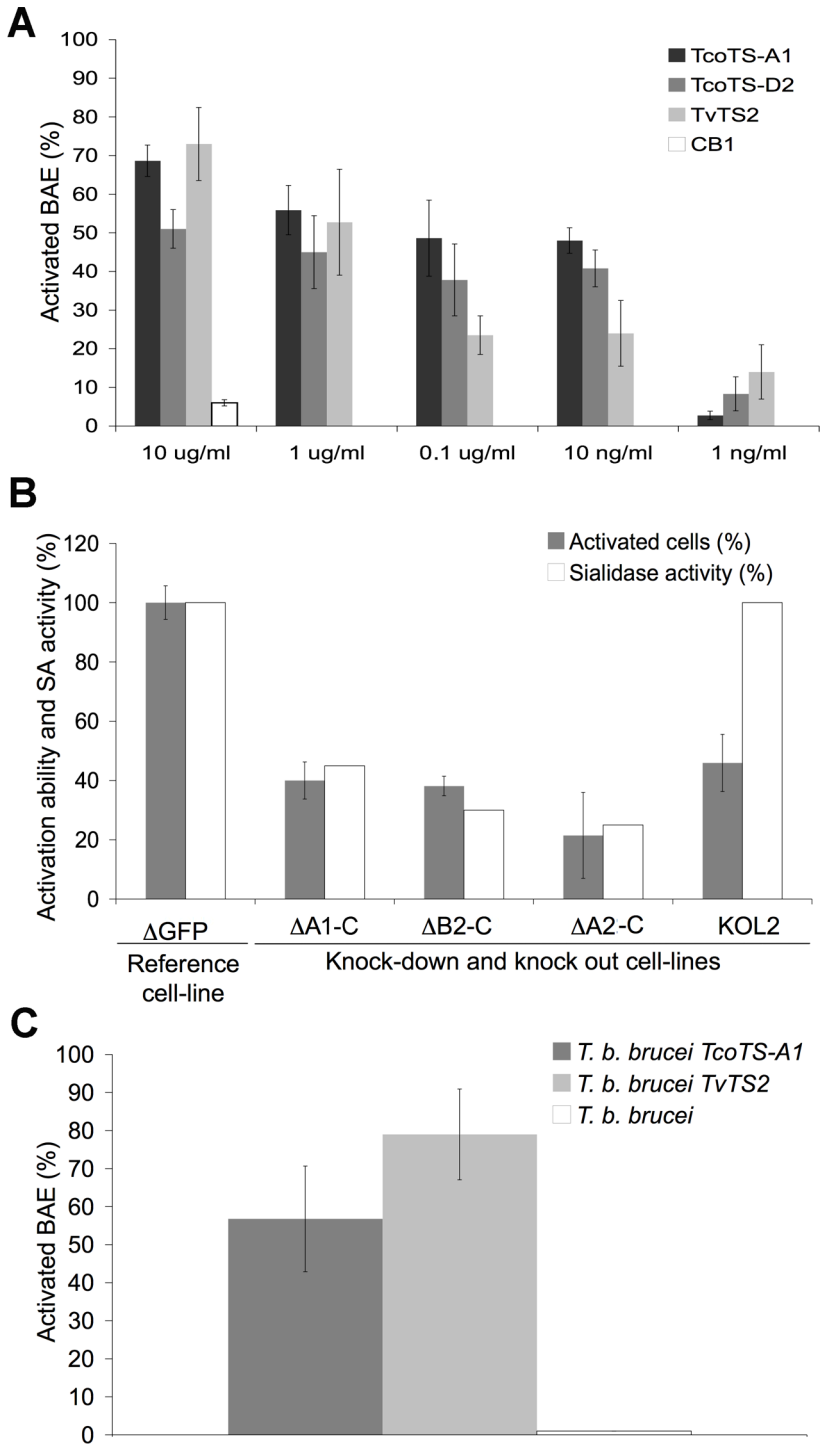
Involvement of trypanosomal TS in BAE activation. (A) Percentage of activated BAE after 16 h of incubation with recombinant TS of *T. congolense* (TcoTS-A1 and D2) and *T. vivax* (TvTS2) or cathepsin CB1 (control recombinant protein). (B) Effects of RNAi silencing and knock out of *T. congolense* TS on BAE activation. Percentage of BAE activated by *T. congolense* IL3000 mutant cell lines ΔA1-C, ΔA2-C, ΔB2-C and KOL2 [21] is normalized to control cell line ΔGFP. Results are represented as percentage of activation capacity. Remaining SA activity in each cell line is indicated as % of the total activity in control cell line. (C) Percentage of activated BAE by *T. b. brucei* 427 expressing heterologous TS of *T. congolense* (*T. b. brucei TcoTS-A1*) or of *T. vivax* (*T. b. brucei TvTS2*) compared to non-transfected cell line. Data are expressed as mean values±SD of three independent experiments. See also Fig. S4.

In order to determine if this activation was mediated by soluble factors released by the trypanosomes, we incubated BAE with i) *T. congolense* or *T. b. gambiense* culture supernatant, ii) *T. congolense* cultured in a transwell: this permeable support insert prevents the physical contact between the two cell types, yet allows the passage of the secreted soluble molecules through a permeable membrane. Results showed that trypanosomes culture supernatant was sufficient to activate BAE (data not shown), and that physical contact was not essential for activation since BAE separated from *T. congolense* by a transwell were still activated (data not shown), which suggests that soluble factors were involved in cell activation. TS are well-known pathogenicity factors in African trypanosomiasis [21], [22], [23] and are involved in endothelial activation by the American trypanosome *T. cruzi* [13]. We previously showed that TS were released from *T. congolense* and *T. vivax* and accumulated in the culture medium during exponential growth [21], [22] but unlike *T. cruzi* [33], these enzymes were released as soluble forms and not into vesicules (data not shown). Consequently, trypanosomal TS could potentially act as soluble mediators of NF-κB activation. Therefore, BAE were incubated for 16 h, separately with three recombinant trypanosomal TS produced in the yeast *Pichia pastoris* as already described [21], [22]: TcoTS-A1, TcoTS-D2 (from *T. congolense*) and TvTS2 (from *T. vivax*) (Fig. 5A). TcoTS-A1, TcoTS-D2 and TvTS2 activated the BAE in a dose dependent manner, while BAE incubated with the recombinant cathepsin CB1 produced and purified according to the same protocol as the TS (control) [34] were not activated. Note that activation of the human and murine cell lines by these recombinant TS showed heterogeneity, similarly to the previous activation results by the African trypanosomes (Table S1).

Moreover, silencing or knock out of TS genes impaired activation capacity of *T. congolense*. Four previously obtained mutant cell lines of *T. congolense* BSF [21] were tested (Fig. 5B). These mutants were less virulent in mice and the induced anaemia is lower [21]. The ΔA1-C cell line contained RNAi constructs designed to be relatively specific to only one subfamily of TS. The ΔA2-C and ΔB2-C cell lines contained constructs with highly conserved sequences shared by all *TcoTS* genes intending to silence active SA and TS. Finally, the knock-out KOL2 cell line was specifically designed to silence *TcoTS-Like2* encoding for an enzymatically inactive TS with a supposedly functional lectin domain. Although activity was not eliminated, an expected decrease in SA activity was observed in the mutant cell lines. The KOL2 cell line displayed 100% SA activity as expected since L2 is an inactive TS. Residual activity in mutant cell lines was expressed as a percentage of total SA activity displayed in the ΔGFP control cell line. Compared to ΔGFP, ΔA1-C, ΔA2-C and ΔB2-C mutant cell lines retained only 40±6.2%, 38±3.3% and 21.5±14.5% of their activation capacity respectively which is consistent with their residual SA activity: 45%, 35% and 25% respectively. Interestingly, the KOL2 cell line also displayed an impaired activation capacity (only 45.9±9.6% of activation capacity retained) (Fig. 5B). This result suggests that catalytic activity may not be required for the activation process.

Furthermore, since no SA nor TS activity was detected in BSF of *T. b. brucei* [21], we overexpressed heterologous TS of *T. congolense* (TcoTS-A1) or *T. vivax* (TvTS2) in *T. b. brucei* BSF to determine the effect of this expression on the activation by this species. Expression of the heterologous TS after induction by tetracycline was confirmed by measuring SA activity in the modified cell lines (Fig. S4). Tetracycline induced *T. b. brucei TcoTS-A1* and *T. b. brucei TvTS2* cell lines activated 56.8±13.9% and 79±11.9% of BAE respectively, compared with 1% of BAE activated by non-transfected *T. b. brucei* cell line (Fig. 5C).

Taken together, these results reflect the prominent role played by trypanosomal TS family in endothelial cell activation.

### Trypanosomal TS induce inflammation *in vivo*

**Figure 6:**
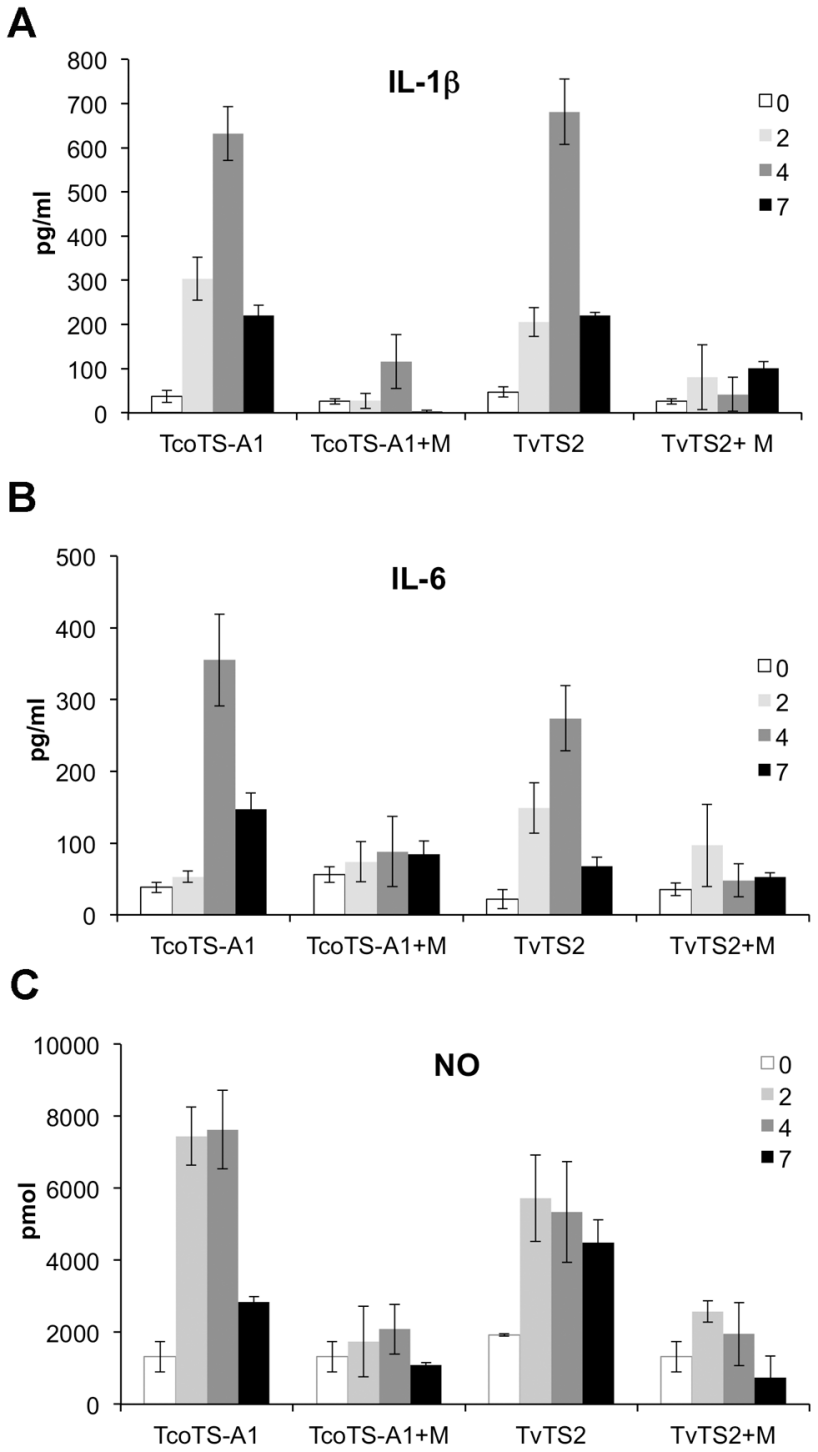
Inflammatory response to TS *in vivo*. Levels of IL-1β (A), IL-6 (B) or total NO (C) were measured at day 0, 2, 4 and 7 in sera of BALB/c mice injected with 100 µg TcoTS-A1 or TvTS2, alone or with myricetin (5 mg/kg). Experiment was repeated twice independently. Data was expressed as mean values±SD of 3 to 6 mice.

To assess the direct effect of TS on inflammation, we injected mice with recombinant TcoTS-A1 or TvTS2 and evaluated the inflammatory response by measuring levels of IL-1β, IL-6 and total NO (nitrate and nitrite) in the sera of mice at days 0 to 7 of the experiment (mice were injected four times on days 0,1,2,4). Injection of TcoTS-A1 resulted in a strong increase of IL-1β, IL-6 and NO levels, most significantly at day 4 (Fig. 6). Compared to pre-injection, IL-1β, IL-6 and NO levels increased approximately 20 fold, 9 fold and 6 fold respectively. Results with TvTS2 were comparable.

When myricetin, a specific inhibitor of trypanosomal TS [22], [35], was injected simultaneously with TS, the inflammation observed in mice was inhibited (Fig. 6). For example, at day 4 after injection, IL-1β levels were 116±60 as opposed to 632±60 pg/ml in the absence of myricetin. This inhibitory effect of myricetin on inflammation is consistent with previous results showing that myricetin impaired TS-induced haematocrit decrease in mice [22].

These results strongly reinforce a direct effect of TS on pathology, and precisely on development of inflammation.

### Identification of TS in *T. b. gambiense* BSF designates these enzymes as a common activation mediator of African trypanosomes

**Figure 7:**
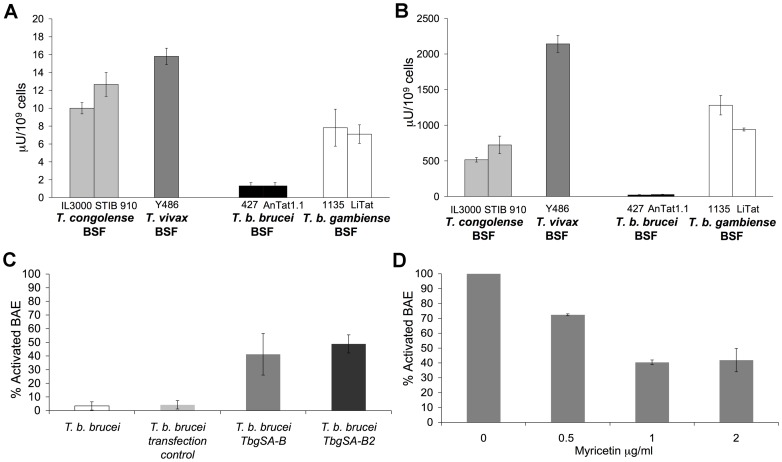
SA and TS activities of *T. b. gambiense* BSF and involvement in BAE activation. (A) SA and (B) TS activities were measured on crude extracts of *T. b. gambiense* 1135 and LiTat BSF and compared with previous results on *T. congolense*, *T. vivax* and *T. b. brucei* BSF [21], [22]. Activities are expressed as nmol of sialic acid released or transferred per min by 10^9^ lysed parasites. (C) Percentage of activated BAE by *T. b. brucei* 427 expressing heterologous TS of *T. b. gambiense T. b. brucei TbgSA B* and *T. b. brucei TbgSA B* compared to non-transfected and transfection control cell lines. (D) Effect of the TS inhibitor myricetin on BAE activation by *T. b. gambiense* 1135 BSF. Results are represented as percentage of activation capacity and normalized to the control (absence of myricetin). Myricetin concentration is indicated on the X-axis. Data are expressed as mean values±SD of three independent experiments. See also Fig. S5 and S6, and Table S2.

Previous studies showed presence of SA activity in BSF of African trypanosomes *T. vivax* and *T. congolense* but not *T. b. brucei* [21], [22]. Interestingly, activation results shown here are consistent with presence of SA activity. Although very close genetically (sequence identity averages 99.2% in coding regions) [36], *T. b. gambiense* and *T. b. brucei* displayed distinct activation profiles. Since no data was available on SA and TS in *T. b. gambiense* BSF, we tested this species for the presence of enzymatic activities. Fluorometric assays revealed both SA and TS activities: 7.82±2.07 µU/10^9^ cells and 1281±137 µU/10^9^ cells of the 1135 strain respectively, and 7.1±1.04 µU/10^9^ cells and 940±21 µU/10^9^ cells of the LiTat strain respectively, compared with almost undetectable activities in *T. b. brucei* BSF (Fig. 7A and B).

In order to identify which enzyme(s) was responsible for this activity in *T. b. gambiense*, and since SA/TS activity was predominantly in the insoluble fraction of *T. congolense* and *T. vivax* [21], mass spectrometry was performed on membrane preparations of *T. b. gambiense* (1135 and LiTat) in comparison with *T. b. brucei* (427 and AnTat 1.1). In both species, peptides belonging to SA genes were identified (Table S2). These genes are well conserved between these two species (Fig. S5). For *T. b. gambiense*, some corresponded to the ortholog of TbTS-like D1 encoded by *Tbg972.2.3310*, others to the ortholog of TbSA B2 encoded by *Tbg.7.8790*, and the rest corresponded commonly to the orthologs of TbSA B encoded by *Tbg972.5.850* and TbSA B2. For *T. b. brucei*, the identified peptides were specific to TbTS-like D1, TbSA B and TbSA B2, encoded by *Tb927.2.5280*, *Tb927.5.640* and *Tb927.7.7480*, respectively. Similarly to *T. congolense*, TS-like enzymes are degenerated and probably not functional. SA B and/or B2 could be active in *T .b. gambiense* BSF but not in *T. b. brucei* BSF, which is consistent with previous results [37] where no enzymatic activity was detected on purified SA B and B2 of *T. b. brucei*. To test this hypothesis, we overexpressed both identified heterologous SA of *T. b. gambiense* in *T. b. brucei* BSF. SA and TS activities of the induced mutant cell lines were verified by fluorometric assays (Fig. S6) and were lower than those of the *T. b. gambiense* BSF WT. As expected, tetracycline induced *T. b. brucei TbgSA B* and *T. b. brucei TbgSA B2* cell lines activated 48.8±6 and 41.2±15.2% of BAE respectively, compared with 3.4±3 and 4.2±3% of BAE activated by the non-transfected and the transfection control *T. b. brucei* cell lines respectively (Fig. 7C). Note that attempts to express *T. b. gambiense* SA B and B2 were not successful in either *E. coli* (solubility problems) or *Pichia pastoris* (absence of expression).

Finally, addition of myricetin, a TS inhibitor, shown to impair TS enzymatic activity and also pathogenicity of *T. vivax* [22], [35], reduced the activation capacity of *T. b. gambiense* 1135 in a dose dependent manner: compared to 100% of activation capacity in absence of myricetin, only 40±1.6% of activation capacity remained in the presence of 1 µg/ml of myricetin (Fig. 7D). Myricetin-induced inhibition of BAE activation by the recombinant TS and by *T. congolense* was also verified (data not shown).

Taken together, these results showed a direct involvement of SA of *T. b. gambiense* in endothelial cell activation, thus making these enzymes a common activation mediator of African trypanosomes.

### Activation is probably mediated by a lectin/sialic acid interaction

**Figure 8:**
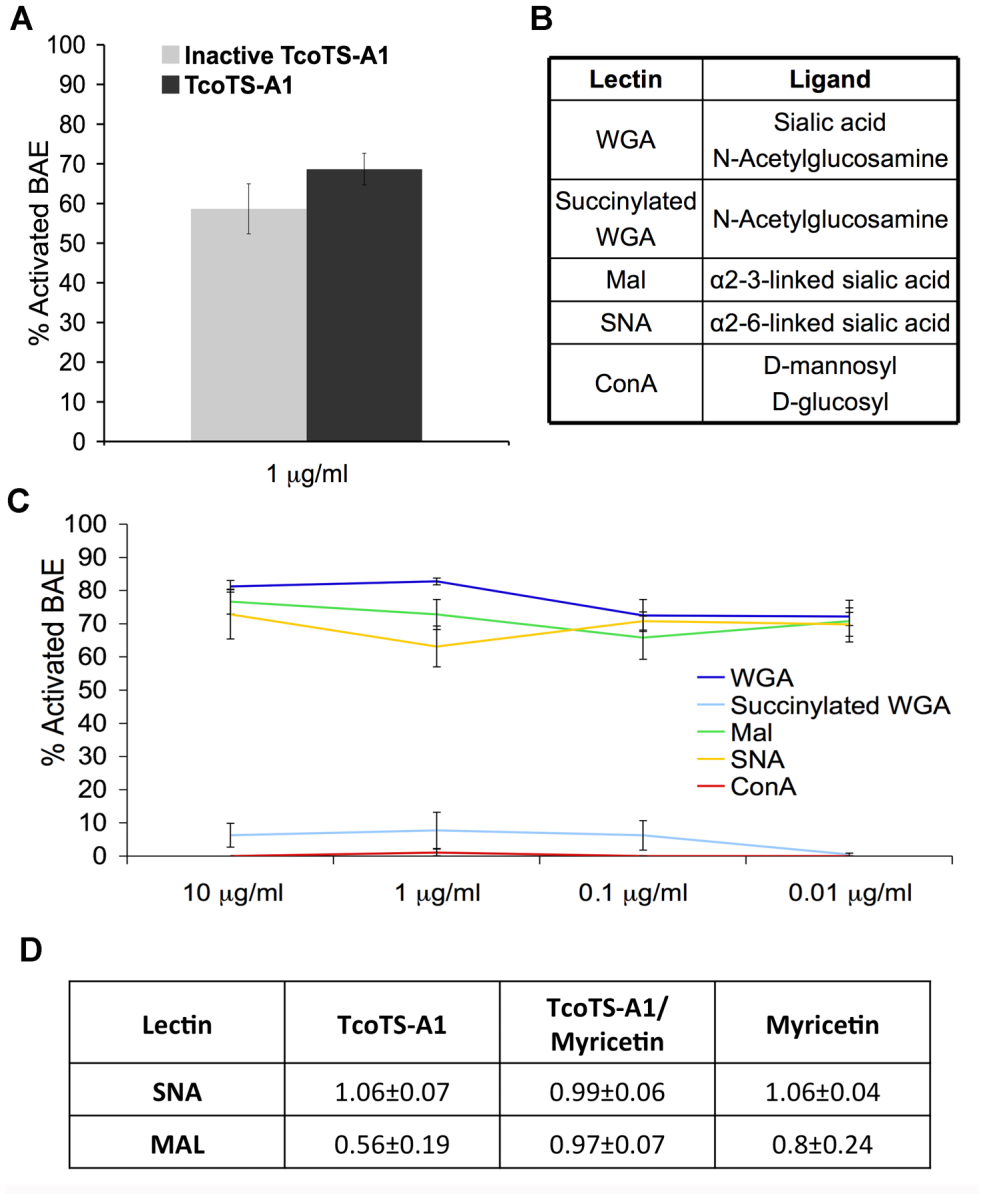
Involvement of TS lectin domain and α-2,3 linked sialic acids in BAE activation. (A) Effect of inactivation of catalytic site of TcoTS-A1 on BAE activation. 10 µg/ml of the active and inactive TcoTS-A1 were incubated for 16 h with BAE. (B) Ligand binding specificity of the lectins. (C) Activation of BAE by the lectins WGA, Mal, SNA, ConA and succinylated WGA after 16 h of incubation. Lectin concentration is indicated on the X-axis. (D) Competitive effect of TcoTS-A1 on MAL binding to BAE. Ratios of median fluorescence intensity (MFI) of BAE in the presence of TcoTS-A1, Myricetin or TcoTS-A1 pre-incubated with myricetin, over MFI of BAE with SNA-FITC or MAL-fluorescein alone. Data are expressed as mean values±SD of three independent experiments.

To further investigate involvement of the lectin domain of TS family members in NFκB activation, we produced a recombinant catalytically inactive mutant of TcoTS-A1 bearing a Tyr^438^: His^438^ substitution with an intact lectin domain, based on the construction of a mutant TS of *T. cruzi*, described previously [13], [38]. Absence of SA and TS activities of the recombinant mutant TS was verified by fluorometric assay (data not shown). Inactivation of the catalytic site had minor effects on the activation capacity of TcoTS-A1, with 58.6±6.3% of BAE activated, compared with 68.6±4% by the active form TcoTS-A1 (Fig. 8A).

BAE cells were also incubated with different lectins. WGA, Mal, and SNA, which recognize sialic acid residues, were able to activate BAE, whereas ConA and succinylated WGA, which have no binding specificity for sialic acid, were not (Fig. 8B and C).

Additionally, desialylation of BAE cell surface by mild periodate treatment inhibited BAE activation by *T. congolense* IL3000 by 47.8±0.1% (data not shown).

Finally, addition of TcoTS-A1 reduced binding of Mal to its α-2,3 sialic acid ligands on BAE to 50% (Fig. 8D), while SNA binding to α-2,6 sialic acids, which is not a substrate for TcoTS-A1 (data not shown) was not affected. Pre-incubation with myricetin abrogated the effect of TcoTS-A1 as a competitor to Mal binding suggesting that myricetin could impair the lectin activity of TcoTS-A1. Myricetin alone had almost no effect on Mal or SNA binding, indicating that its inhibition of TcoTS-A1 lectin binding and subsequently activation of endothelial cells is indirect, possibly due to a steric hindrance after binding to the catalytic domain.

These results indicated that the process of endothelial cell activation involves the lectin domain of trypanosomal TS and most likely α-2,3 linked sialic acid on endothelial cell surface.

## Discussion

Host-pathogen interaction in AAT and HAT is poorly understood. As parasite strains resistant to chemotherapy arise [17], the needs for accurate knowledge of this interaction are increasing, mainly to move forward to an anti-disease strategy, a promising method of controlling trypanosomiasis. During infection by African trypanosomes, an intimate interaction with the host endothelium occurs, and exploring it can provide considerable insights on the host-pathogen interaction.

Using indirect immunofluorescence, we showed that *T. congolense*, *T. vivax* and *T. b. gambiense* were capable of activating endothelial cells through the NF-κB pathway, and more specifically the classical NF-κB pathway. In fact, immunofluorescent studies were conducted using anti-p65 antibodies, and inhibition assays using JSH-23 specific inhibitor of p65 subunit of NF-κB, a subunit implicated in the classical NF-κB pathway only. We also established that the Ser 32/36 of IκBα is phosphorylated, which is another characteristic of the classical NF-κB activation pathway [9], and further implies an involvement of IKKβ, a kinase known to play a critical role in this specific phosphorylation.

Endothelial cell activation by the African trypanosomes was species and tissue specific, and each endothelial cell line displayed a specific pattern of activation kinetics. These findings are consistent with the well-known phenotypic variation between endothelial cells from different species and, moreover, from different locations within the same species [7]. Interestingly, the tissue specificity observed with *T. congolense* activation is consistent with the tropism to lung, bone marrow and peripheral lymph nodes observed *in vivo* [5]. Nevertheless, the observed species-specific activation of endothelial cells by *T. congolense* remains to be elucidated. It may possibly be linked to a TS binding specificity given that endothelial glycocalyx presents vast differences among microvascular endothelial cells of different species [30]. On the other hand, some cell lines were activated by the parasites but not the recombinant TS nor lectins. This can be explained by a default of enzyme's accessibility to substrate, compared to a parasite that releases numerous molecules, such as proteases, which can expose specific substrates, making them more accessible.

Using species and organ specific endothelial cell lines seemed an effective strategy to avoid overlooking specific features of the trypanosome/endothelial cell interaction due to this phenotypic variation. This should be taken into consideration when selecting endothelial cell lines adapted for trypanosomiasis studies.

Several arguments supported a potential role of TS in endothelial cell activation, in addition to their key role in infection and anaemia in AAT [21], [22]. The fact that *T. congolense* and *T. vivax* BSF release these enzymes into the blood *in vivo* and into the culture media *in vitro* [23], makes TS potential soluble mediators of the interaction process. We consequently demonstrated this role by showing that recombinant trypanosomal TS activated the BAE, and that inhibition of expression of TS impaired the activation capacity of *T. congolense* mutant cell lines.

Significantly, trypanosome species that activated the endothelial cells possess SA activity in their BSF whereas *T. b. brucei*, which is incapable of endothelial activation, do not. Furthermore, over-expression of heterologous TS of *T. congolense* and *T. vivax* enabled *T. b. brucei* BSF to activate BAE. The controversial finding that *T. b. brucei* did not activate any of the endothelial cell lines, even though the closely related subspecies *T. b. gambiense* did, could be explained by the fact that only *T. b. gambiense* BSF expressed active TS. Here we detected SA and TS activities in *T. b. gambiense* BSF and used mass spectrometry to identify peptides corresponding to orthologs of TbTS like-D1, TbSA B2 and TbSA B. However we cannot entirely exclude the possibility of presence of TbTS ortholog in *T. b. gambiense* even though no corresponding peptides were found. Note that this protein was detected by the same technique in the procyclic forms of the parasite, as expected. Surprisingly, peptides corresponding to these same three enzymes were identified in *T. b. brucei* BSF. Even though expressed in both species, these SA seem to be enzymatically active only in *T. b. gambiense*. Only four amino acids differ between TbSA B2 and TbgSA B2 (Fig. S4), and three between TbTS-like D1 and its ortholog whereas there are significantly more differences between TbSA B and TbgSA B. These substitutions are mostly in the lectin domain which could, therefore, affect sugar binding, and subsequently impair the SA/TS enzymatic activity and the activation capacity of *T. b. brucei*. This is consistent with previous studies describing punctual substitutions with major impact on TS activity [38], [39]. This raises the question about the suitability of *T. b. brucei* as a model for the study of *T. b. gambiense-* caused HAT.

These results strongly confirm the involvement of TS in endothelial cell activation, but above all, they suggest that TS represent a common mediator of endothelial cell activation among trypanosome species that induce divergent physiopathologies.

More specifically, the lectin-like domain of the TS appeared to be predominantly responsible for the endothelial cell activation. Firstly, the knockout of *TcoTS-Like2* impaired the activation of BAE by *T. congolense* and secondly the loss of TS enzymatic activity by inactivating the catalytic site had no effect on the BAE activation. Additionally, commercial lectins recognizing sialic acid residues on the endothelial cell surface activated the BAE, which not only reinforces our results on lectin domain involvement but also, and together with the finding that desialylation of BAE surface inhibits their activation, points towards an implication of sialic acid in this trypanosome/endothelial cell interaction. TcoTS-A1 selectively inhibited the binding of MAL on BAE indicating that α-2,3 sialylated molecules act as potential receptors of TS on the endothelial cell surface.

In conclusion, the endothelial cell activation is apparently mediated by α-2,3 sialylated receptors on the endothelial cell surface, and TS of the trypanosome, most likely through their lectin domain. This result is similar in *T. cruzi*-mediated endothelial cells activation [13] but as in *T. cruzi*, molecules bearing such sialic acids residues have not been yet identified.

To our knowledge, this would be the first report showing a direct evidence of NFκB activation by lectins. NFκB activation by neuraminidase through sialidase activity is well known. It is mostly described for endogenous mammalian SA such as Neu1 desialylation of TLR4 [40]. It was also demonstrated with pathogen SA such as NanA of *Streptococcus pneumoniae* [41]. However activation by pathogen lectins is very poorly described. Only one study has suggested that streptococci stimulation of endothelial cells and subsequent release of cytokines involved lectin interactions [42]. Interestingly, our evidence correlates with the role of inactive TS of *T. cruzi* in endothelial cell activation [13], which could suggest lectin involvement, thus promoting a novel role of lectin binding domains of trypanosomal TS in host/pathogen interactions.

NF-κB activation is known to impact on host/pathogen relationship in parasitic infections, in particular by regulating immunity and inflammation [11]. Knowing that SA/TS silencing correlates with impairment of virulence in experimental infection with *T. congolense* [21], and that recombinant SA/TS directly contribute to anaemia by erythrocyte desialylation and subsequent erythrophagocytosis, it was crucial to elucidate their role in inflammation. Here, we showed that NF-κB activation of endothelial cells induced a pro-inflammatory response *in vitro* shown by the production of nitric oxide via the NO synthase, of the pro-inflammatory cytokines IL-1β and IL-6, and the expression of the adhesion molecules ICAM-1 and VCAM-1, known to intervene in leukocyte recruitment during infections. Most importantly, our results demonstrate that TS alone can induce a pro-inflammatory response in mice.

This production of pro-inflammatory molecules could have significant consequences because it participates in the cascade of events leading to inflammation, a phenomenon that is closely related to AAT clinical symptoms and more specifically cachexia and anaemia, known as the major lethal features of AAT contributing to mortality. For instance, it has been demonstrated that NO can be implicated in host pathology by inhibiting hematopoiesis [43]. Anaemia can also be partially linked to inflammation via non-specific erythrophagocytosis by macrophages hyperactivated by chronic inflammation [44], and also via the IL-6 induced production of hepcidin, an iron regulatory hormone [45]. Additionally, cachexia is mainly a consequence of excessive production of TNFα [46] which is a major inflammatory cytokine. On the other hand, the late stages of HAT are characterized by parasites crossing the BBB and invasion of the CNS [3], [4], [47] by mechanisms that are still not fully understood. One proposed model is that parasites could use leukocytes alteration to disrupt the integrity of the endothelial barrier to penetrate the nervous system [48]. Here we show a NF-κB dependent induction of ICAM1 and VCAM1 expression. These are crucial adhesion molecules in the process of leukocyte transmigration, therefore, NFκB activation by *T. b. gambiense* may potentially play a role in crossing the BBB, by increasing leukocyte migration and subsequently, parasite penetration into the CNS. However, TS presence does not necessarily imply BBB crossing, which probably involves other factors.

**Figure 9:**
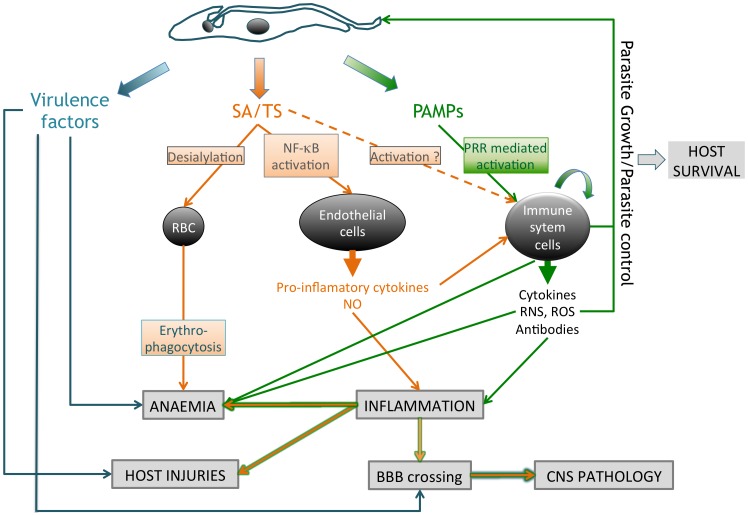
Multifunctional role of trypanosomal SA/TS in host/pathogen molecular crosstalk. ROS: Reactive Oxygen Species, RNS: Reactive Nitrogen Species, PAMPs: Pathogens Associated Molecular Patterns, PRR: Pathogen Recognition Receptor.

In summary, this work reinforces the role of TS as virulence factors in AT, and upholds the hypothesis that TS-induced NF-κB activation may contribute to inflammation, an important component in the development of pathogenesis during AT. Eventually, the already established anti-disease effect of immunization by TS in mice [21] may be partially explained by its effect on the endothelial activation and the consequent inflammatory response. Here, we proposed a dual role for trypanosomal SA/TS in both inflammation and anemia (Fig. 9). In fact, SA/TS are released very early in the infection course and cause erythrocyte desialylation, strongly contributing to the development of anaemia at least in the acute phase of the infection; at the same time, SA/TS activate endothelial cells leading to a pro-inflammatory response with outcomes on the immune system, anaemia development and potentially on BBB crossing [49]. SA/TS might also participate in immune system activation as described in *T. cruzi* [50]. Other virulence factors are well known to intervene in this host/pathogen interaction, such as cathepsins and PAMPs (Pathogen-Associated Molecular Patterns). Cathepsins cause host injuries, anaemia and could participate in BBB crossing [51]. PAMPS, like CpG DNA, GPI-anchor of VSG, etc., interact with specific receptors, called PRR (Pattern Recognition Receptors) and trigger a host immune response leading to production of cytokines and other mediators that participate in inflammation, anaemia and host injuries [52], [53]. Finally, all these factors sustain a balance between parasite growth and its control, on which the host survival depends. Here we propose a model of the host/pathogen molecular cross talk showing the central role of SA/TS. In such a model, parasites use different strategies to trigger anaemia and inflammation. This configuration is consistent with previous results showing that mice models respond differently to infections depending on the trypanosome species [54]. It also explains why in field infections, the severity of the disease and its physiopathological features are widely dependent on the trypanosome species and its specificities, such as the ability to invade tissues, the genetic variability between isolates (especially from distinct geographical regions) and above all the host range [55].

This study provides a considerable background to identify the cellular components responsible for the interaction of African trypanosomes with endothelial cells, which represents a central part of the understanding of the host/pathogen molecular cross talk. The functional importance of the endothelial cell activation needs to be further explored, mainly by *in vivo* studies in field infections comparing the inflammatory responses of mammalian hosts to African trypanosomes species with different capacities for endothelial cell activation *in vitro*.

Lastly, this work defines TS as a promising new target for an anti-disease strategy to control trypanosomiasis in Africa, especially as TS represent a common interaction molecule for both human and animal African trypanosomes.

## Materials and Methods

### Ethics statement

All animal procedures were carried out in strict accordance with the French legislation (Rural Code articles L 214-1 to L 214-122 and associated penal consequences) and European Economic Community guidelines (86-6091 EEC) for the care of laboratory animals and were approved by the Ethical Committee of Centre National de la Recherche Scientifique (CEEA50), Région Aquitaine and by the University of Bordeaux 2 animal care and use committee (Permit number: A33-063-916). All efforts were made to minimize animal suffering.

### Recombinant TS injections in mice

Eight-week-old female BALB/c mice were injected intraperitoneally with 100 µg of recombinant trypanosomal TS, named TcoTS-A1 and TvTS2 and previously described [21], [22], for 3 consecutive days (days 0,1 and 2) and a fourth time on day 4, either alone or with myricetin (Sigma, 5 mg kg^−1^). Blood samples were collected on day 0, 2, 4, and 7 by tail bleed in 100 µl capillary tubes coated with Na-heparin and serum was prepared for further analyses.

### Trypanosome culture

Bloodstream forms (BSF) of the *T. congolense* IL3000 or STIB910 strain (kindly provided by the International Livestock Research Institute, Nairobi, Kenya) and genetically modified derivatives were cultivated as previously described [56]. BSF of the *T. vivax* Y486 strain (isolated in Nigeria and kindly provided by the International Livestock Research Institute, Nairobi, Kenya) were collected from infected mice blood as described [22] and maintained in Tc BSF medium supplemented with 20% GS and 2 mM glutamine. BSF of the *T. b. gambiense* 1135 or LiTat strain [57] and of the *T. b. brucei* AnTat 1.1 or 427 strain were maintained in IMDM (Fisher) supplemented with 10% FCS and 150 mM L-cystein (Sigma).

### Endothelial cell culture

Primary cultures of bovine aortic endothelial cells (BAE) (kindly provided by E. Genot, Bordeaux) were cultured in Tc BSF specific medium [56] supplemented with 20% GS (goat serum, Invitrogen) and 2 mM glutamine (Sigma), and used between passages 5 and 15. Human endothelial cell lines from umbilical vein, brain, peripheral lymph nodes, lung, intestine, skin, appendix, and murine endothelial cell line from brain, lung, peripheral lymph nodes, spleen, thymus, and bone marrow were kindly provided by C. Kieda (Orléans, France) and were cultured in Opti MEM I medium (Invitrogen) supplemented with 2% FCS (fetal calf serum), 0.4% penicillin/streptomycin (Invitrogen), and 0.2% fungizone, in 24 well plates in a 5% CO_2_ humidified atmosphere at 37°C. For coculture experiments, the seeded endothelial cells were washed in PBS (phosphate-buffered saline: 137 mM NaCl, 10 mM Phosphate, 2.7 mM KCL, pH 7.4), maintained in Tc BSF without serum for 24 hours, then cocultured with *T. congolense*, *T. b. brucei*, *T. b. gambiense* (10^6^/ml) or *T. vivax* (10^5^/ml) in Tc BSF medium supplemented with 10% GS. Culture with recombinant proteins or lectins was performed in DMEM supplemented with 1% Serum + (Invitrogen), 1 mM sodium pyruvate (Invitrogen), and 50 UI/ml/50 µg/ml penicillin/streptomycin.

### Construction of *T. congolense* and *T. b. brucei* mutant cell lines

*T. congolense* mutant cell lines ΔA1-C, ΔB2-C, ΔA2-C (knock down of TS by RNAi), ΔGFP (control cell line) and KOL2 (knock out of *TcoTS-Like2* gene) were previously obtained in the laboratory [21]. Briefly, RNAi transfections were performed in IL3000 for constitutive expression of the transgene. Transfected cell lines containing the three constructs p2T7^Ti^-A1-C, p2T7^Ti^-B2-C and pLew100-A2-C were named ΔA1-C, ΔB2-C and ΔA2-C respectively. As a control of transfection and experimental infection, the p2T7^Ti^/GFP vector (kindly provided by J.E. Donelson) [58] was inserted into the IL3000 strain. For the knockout cell line, IL3000 was transfected with pGLneo-TcoTS-Like2 and subsequently with pGLbla-TcoTS-Like2 to obtain the double knockout cell line named KOL2.

For expression of heterologous sialidases in BSF of *T. b. brucei*, genes encoding TcoTS-A1, TvTS2, TbgSA B and TbgSA B2 were cloned in the plew100 vector then transfected in the *T b. brucei* 427:90-13 BSF strain using Amaxa system with the X001 program as described by the manufacturer. Transfectants were selected by addition of phleomycine (2.5 µg/ml) in the culture medium, and checked by PCR on genomic DNA. Expression of the transgene was induced by addition of tetracycline (1 µg/ml) in the culture medium. The vector plew100 containing GFP was used as transfection control.

### Expression of recombinant TS in *Pichia pastoris* and protein purification

Mutant inactive recombinant TcoTS-A1 of *T. congolense* was constructed by site-directed mutagenesis (using QuikChange II; Stratagene) using specific mutant primers bearing a Tyr^438^ :His^438^ substitution and expressed in the EasySelect *Pichia* system (Invitrogen) according to the manufacturer's instructions. Expression in the *Pichia pastoris* strain X-33 and subsequent purification using ion-exchange chromatography were performed as described previously [21].

The recombinant proteins TcoTS-A1, TcoTS-D2, TvTS2 and CB1 were previously obtained in the laboratory [21], [22], [34]

### Indirect immunofluorescence

Endothelial cells were grown on glass slides into 24 wells plate and incubated with BSF of the wild type and mutant cell lines of *T. congolense* IL3000 and STIB910 strains, *T. b. brucei* AnTat 1.1 and 427 strains, *T. b. gambiense* 1135 and LiTat strains, *T. vivax* Y486 strain, and different amounts of the recombinant sialidases or the commercial lectins SNA, Mal II, WGA, ConA (GaLab) and succinylated WGA (Vector Laboratories). At different time points, cells were washed in PBS, fixed with 360 µl paraformaldehyde (1% in PBS) for 10 min, permeabilized with 40 µl Triton X-100 (1% in PBS) for 5 min, and PFA was neutralized by adding 40 µl glycin (1 M in PBS) for 5 min. Cells were then washed twice in PBS and slides were incubated with rabbit polyclonal anti-NF-κB antibody (Cell Signaling) (1∶100 in PBS 0.1% triton X-100 0.1% BSA) for 40 min, washed, then incubated for 20 min with Alexa Fluor 488 mouse anti-rabbit IgG antibody (Molecular Probes) (1∶100 in PBS 0.1% Triton X-100 0.1% BSA). DNA was stained by adding 1 µg/ml 4′,6′-diamidino-2-phenylindle (DAPI) for 5 min, and slides were mounted in Vectashield (Vector Laboratories). Cells were observed with a Zeiss UV microscope and images were captured using a MicroMax-1300Y/HS camera (Princeton Instruments) and Metamorph software (Universal Imaging Corporation).

### Cytokine measurement

Interleukins IL-1β and IL-6 were measured in 100 µl of mice sera for *in vivo* assays, or 100 µl of supernatant from a 2 h, 6 h, 16 h, 24 h and 48 h *in vitro* coculture of M Lung or M Bone Marrow endothelial cell lines with *T. congolense* IL3000 strain or *T. b. brucei* AnTat 1∶1 strain. Assays were performed using the ELISA Ready-SET-Go kits (eBioscience) according to the manufacturer's instructions.

### NO assay

For *in vitro* assays, M Lung and M Bone Marrow cell lines, or BAE were incubated for 6 h, 16 h and 24 h with *T. congolense* IL3000 strain and washed in PBS. Generation of intracellular nitrite was determined by adding 5 µM of 5,6 diaminofluorescein diacetate (DAF-2DA, Sigma) for 40 min at 37°C in the dark. Medium was removed, cells were washed in PBS for 15 min, and fluorescence intensity was measured with an Optima plate reader (BMG Labtech, Germany) (Excitation/Emission: 485/520). This assay was also performed after addition of 1 mM Nω-Nitro-L-arginine (Sigma), a competitive inhibitor of NO synthase. For *in vivo* assays, total nitrate and nitrite levels in mice sera were measured using a fluorometric kit (Calbiochem) according to the manufacturer's instructions. Sera were deproteinized by ultrafiltration through Vivaspin columns (Sartorius) prior to assay. Fluorescence intensity was measured with an Optima plate reader (BMG Labtech, Germany) (Excitation/Emission: 350/420).

### Flow cytometry analysis of adhesion molecules expression

M Lung and M Bone Marrow endothelial cell lines were cocultured with *T. congolense* for 6, 16 and 24 h. Culture medium was removed and cells washed then detached by trypsin treatment. Endothelial cells Fc receptors were blocked by adding mouse serum (1∶10) for 30 min. Cells were then incubated with rat monoclonal antibody anti-CD31 APC conjugated (eBioscience) and rat mAb anti-ICAM1 or anti-VCAM1 FITC conjugated (Biolegend) for 30 min on ice, then washed in PBS. Data were collected on a FACS Canto (Becton Dickinson) flow cytometer and analysed using the FACSDiva software. Cells were gated for forward and side-angle scatters and 10 000 fluorescent particles of each gated population were analysed. The analytical method was described in the results.

### Flow cytometry analysis of lectin binding to endothelial cells

BAE layers were detached with a cell scraper, washed with PBS and incubated with FITC conjugated SNA (GaLab) (10 µg/ml in PBS 1% BSA) or fluorescein conjugated MAL (Vector Laboratories) (10 µg/ml in PBS 1% BSA) for 30 min on ice. For the lectin inhibition binding assay, BAE were incubated with 10 µg TcoTS-A1, 0.5 µg Myricetin (Sigma) or TcoTS-A1 pre-incubated with Myricetin (10 µg∶0.5 µg).

### SDS-PAGE and immunoblotting

Total protein preparations of M Lung (one confluent well of a 6 wells plate per condition) were obtained by adding 100 µl of 1% SDS with loading buffer and heating at 100°C in the presence of a protease inhibitor cocktail (complete Mini, EDTA-free; Roche Diagnostics, GmbH). 20 µl were loaded per well and separated by SDS-PAGE (10%) before transfer onto polyvinyl difluoride (PVDF) membranes (Immobilon-P, Millipore). Membranes were incubated with mouse monoclonal anti-phospho-IκB-α (Ser32/36) or anti-IκB-α (Cell Signaling) 1∶1000 in TBS (Tris-buffered saline: 50 mM Tris, 150 mM NaCl, pH 7.6) 0.05% tween20, 5% BSA followed by horseradish peroxidase -conjugated anti-mouse immunoglobulin G (IgG) (Sigma; 1∶10 000). Antigen-antibody interactions were developed using Immobilon Western chemiluminescent HRP substrate (Millipore).

### Sialidase and trans-sialidase activity assays

Sialidase (SA) and trans-sialidase (TS) activities were determined as previously described [59]. Details are provided in Text S1.

### Desialylation of BAE by mild periodate treatment

To desialylate BAE, cells were treated with mild periodate, which specifically destroys the glycerol side chain (C7–C9) of sialic acids, as previously described [60], [61]. BAE were incubated with 2 mM NaIO_4_ solution for 30 min at 37°C. BAE viability was not affected by this treatment.

### Mass spectrometry on membrane preparations

Membrane preparations of *T. b. gambiense* 1135 or LiTat BSF or *T. b. brucei* 427 BSF, SDS-PAGE and mass spectrometry analysis were performed as described previously [21]. Details are provided in Text S1.

## Supporting Information

Figure S1 **Related to** **Figure 2** **. Activation of murine and human endothelial cells by TNFα or** ***T. b. brucei*** **AnTat 1.1 BSF.** Percentage of activated (A and C) murine (M) and (B and D) human (H) endothelial cells is determined after 4 h of incubation with 5 ng/ml of TNFα (A and B) and after 2, 6, 16 and 24 h of coculture with *T. b. brucei* AnTat 1.1 (C and D). Results were similar with *T. b. brucei* 427 strain. Data are expressed as mean values ±SD of three independent experiments (C and D).
(TIF)

Figure S2 **Related to** **Figure 2** **. NF-κB immunofluorescent staining in murine and human endothelial cell lines after 24 h of coculture with** ***T. congolense*** **IL3000.** Results were similar with *T. congolense* STIB910 strain.
(TIF)

Figure S3 **Related to** **Figure 3** **. Absence of IκBα phosphorylation and pro-inflammatory response of endothelial cells cocultured with** ***T. b. brucei*** **AnTat 1.1.** (A) Secretion of IL-1β and IL-6 by M Bone Marrow (BM) (left) and M Lung (right) microvascular endothelial cells in the supernatant after 6 and 24 h of coculture with BSF of *T. b. brucei* AnTat 1.1. (B) Expression of VCAM-1 and ICAM-1 adhesion molecules on the surface of M Lung and M BM microvascular endothelial cells after 24 h of coculture with *T. b. brucei* AnTat 1.1. Ratios of MFIs (obtained by flow cytometry analysis) were calculated as detailed in Materials and methods. (C) Absence of phosphorylated IκBα by immunoblotting. M Lung were cultivated with *T. b. brucei* AnTat 1.1 for 0, 1, 2, 4 or 8 h and total protein extracts were subjected to western blotting with anti IκBα (upper panel), anti phospho-IκBα (median panel) or anti-tubuline (lower panel) antibodies.
(TIF)

Figure S4 **Related to** **Figure 5** **. Overexpression of heterologous TcoTS-A1 and TvTS2 in** ***T. b. brucei*** **BSF.** SA activity was measured on crude extracts of *T. b. brucei TcoTS-A1* and *T. b. brucei TvTS2* cell lines and compared to the non-transfected strain *T. b. brucei* 427 BSF. Data are expressed as mean of 3 values measured on two independent experiments.
(TIF)

Figure S5 **Related to** **Figure 7** **. SA and TS genes in** ***T. b. brucei*** **and** ***T. b. gambiense*** **genomes.** (A) List of the *T. b. brucei* SA and TS genes was established from genome database (http://tritrypdb.org/tritrypdb/) and from two previous studies on these genes [37], [39]. *T. b. gambiense* orthologs were found in genome database (http://tritrypdb.org/tritrypdb/). (B) Sequence alignment of SA B and SA B2 of *T. b. gambiense* and *T. b. brucei*.
(TIF)

Figure S6 **Related to** **Figure 7** **. Overexpression of heterologous TbgSA B and TbgSA B2 in** ***T. b. brucei*** **BSF.** SA and TS activities were measured in crude extracts of *T. b. brucei TbgSA B* and *T. b. brucei TbgSA B2* cell lines and compared to the non-transfected cell line *T. b. brucei* 427 BSF.
(TIF)

Table S1 **Related to** **Figure 2** **. Heterogeneity of endothelial cell activation by African trypanosomes and recombinant TS.** ^a^ M lung, M BM, M spleen, M brain, M thymus. ^b^ HUVEC, H lung, H brain, H skin, H intestine, H appendix.
(DOC)

Table S2 **Related to** **Figure 7** **. Identification of SA or TS expressed in** ***T. b. brucei*** **and** ***T. b. gambiense*** **BSF by mass spectrometry.** (A) Sialidase peptides in membrane preparations of *T. b. gambiense* 1135 and LiTat BSF. TbgTS-LikeD1, TbgSA-B and TbgSA-B2 are encoded by Tbg972.2.3310, Tbg972.5.850 and Tbg.7.8790 respectively. (B) Sialidase peptides in membrane preparations of *T. b. brucei* 427 BSF.
(DOC)

Text S1 **Supplemental experimental procedures.**
(DOC)
