# Supplementary figures and images for: Identification of Trans-Sialidases as a Common Mediator of Endothelial Cell Activation by African Trypanosomes
Source: PLoS Pathog. 2013 Oct 10;9(10):e1003710. doi: 10.1371/journal.ppat.1003710 (PMC3795030; doi:10.1371/journal.ppat.1003710)

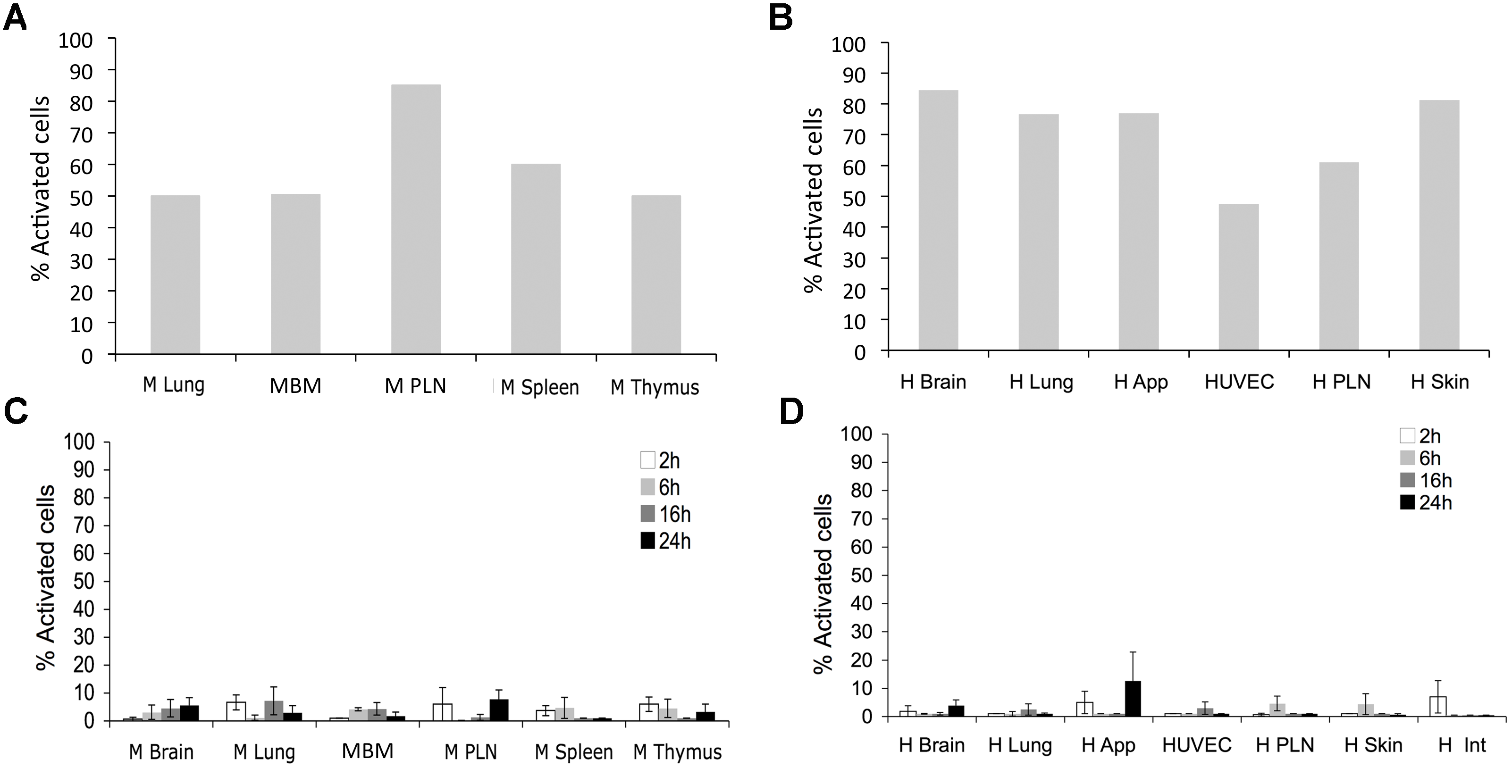

Supplement: Figure S1 — Related to Figure 2 . Activation of murine and human endothelial cells by TNFα or T. b. brucei AnTat 1.1 BSF. Percentage of activated (A and C) murine (M) and (B and D) human (H) endothelial cells is determined after 4 h of incubation with 5 ng/ml of TNFα (A and B) and after 2, 6, 16 and 24 h of coculture with T. b. brucei AnTat 1.1 (C and D). Results were similar with T. b. brucei 427 strain. Data are expressed as mean values ±SD of three independent experiments (C and D). (TIF) [file ppat.1003710.s001.tif]

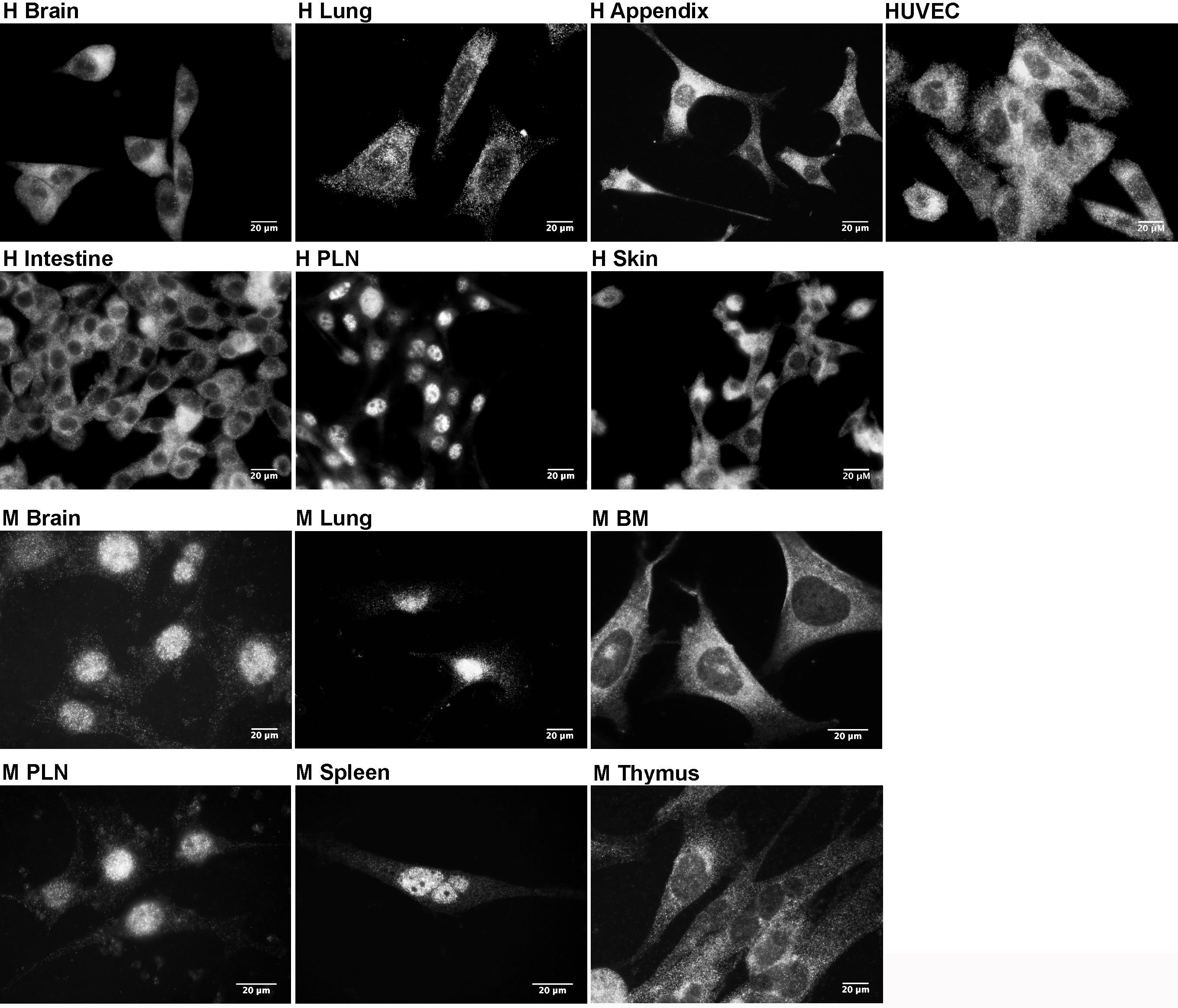

Supplement: Figure S2 — Related to Figure 2 . NF-κB immunofluorescent staining in murine and human endothelial cell lines after 24 h of coculture with T. congolense IL3000. Results were similar with T. congolense STIB910 strain. (TIF) [file ppat.1003710.s002.tif]

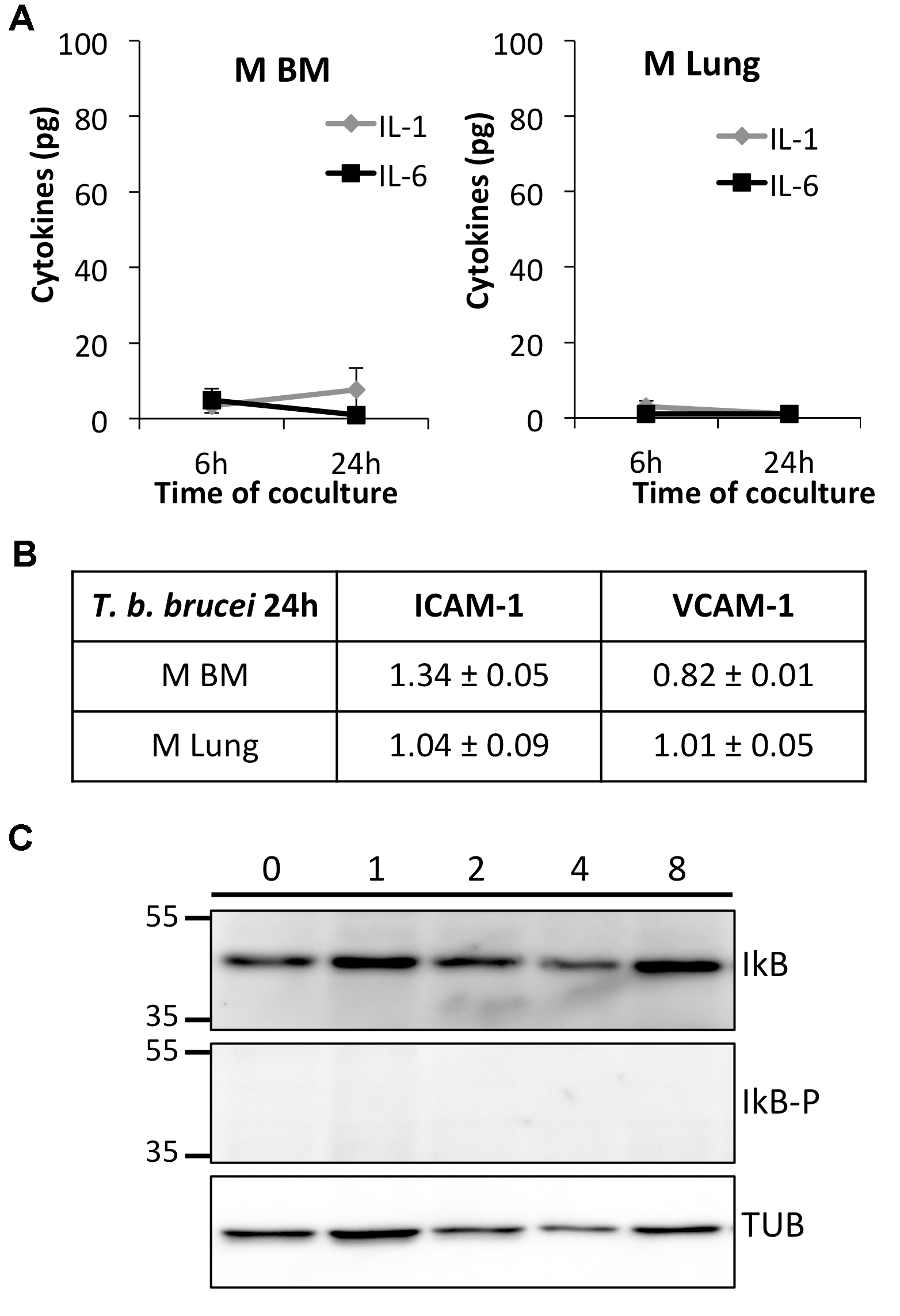

Supplement: Figure S3 — Related to Figure 3 . Absence of IκBα phosphorylation and pro-inflammatory response of endothelial cells cocultured with T. b. brucei AnTat 1.1. (A) Secretion of IL-1β and IL-6 by M Bone Marrow (BM) (left) and M Lung (right) microvascular endothelial cells in the supernatant after 6 and 24 h of coculture with BSF of T. b. brucei AnTat 1.1. (B) Expression of VCAM-1 and ICAM-1 adhesion molecules on the surface of M Lung and M BM microvascular endothelial cells after 24 h of coculture with T. b. brucei AnTat 1.1. Ratios of MFIs (obtained by flow cytometry analysis) were calculated as detailed in Materials and methods. (C) Absence of phosphorylated IκBα by immunoblotting. M Lung were cultivated with T. b. brucei AnTat 1.1 for 0, 1, 2, 4 or 8 h and total protein extracts were subjected to western blotting with anti IκBα (upper panel), anti phospho-IκBα (median panel) or anti-tubuline (lower panel) antibodies. (TIF) [file ppat.1003710.s003.tif]

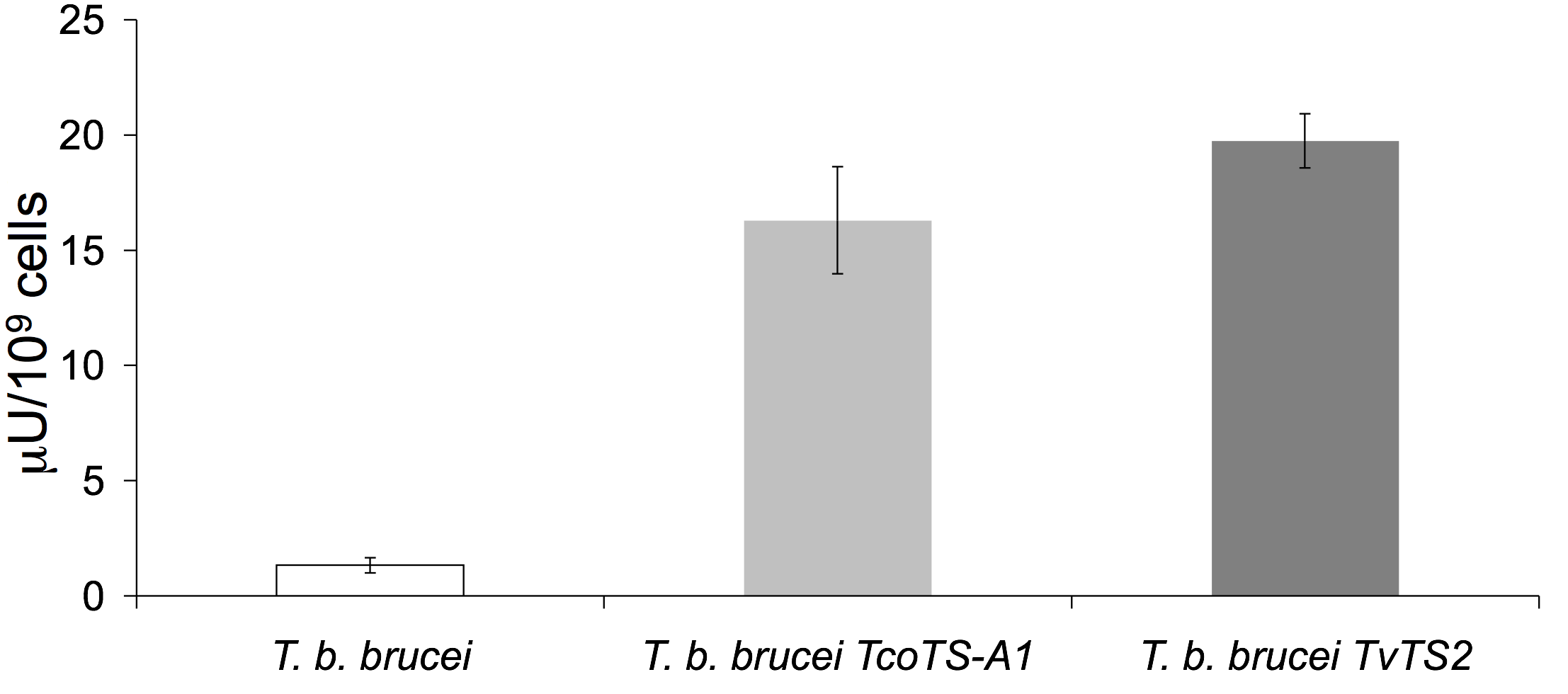

Supplement: Figure S4 — Related to Figure 5 . Overexpression of heterologous TcoTS-A1 and TvTS2 in T. b. brucei BSF. SA activity was measured on crude extracts of T. b. brucei TcoTS-A1 and T. b. brucei TvTS2 cell lines and compared to the non-transfected strain T. b. brucei 427 BSF. Data are expressed as mean of 3 values measured on two independent experiments. (TIF) [file ppat.1003710.s004.tif]

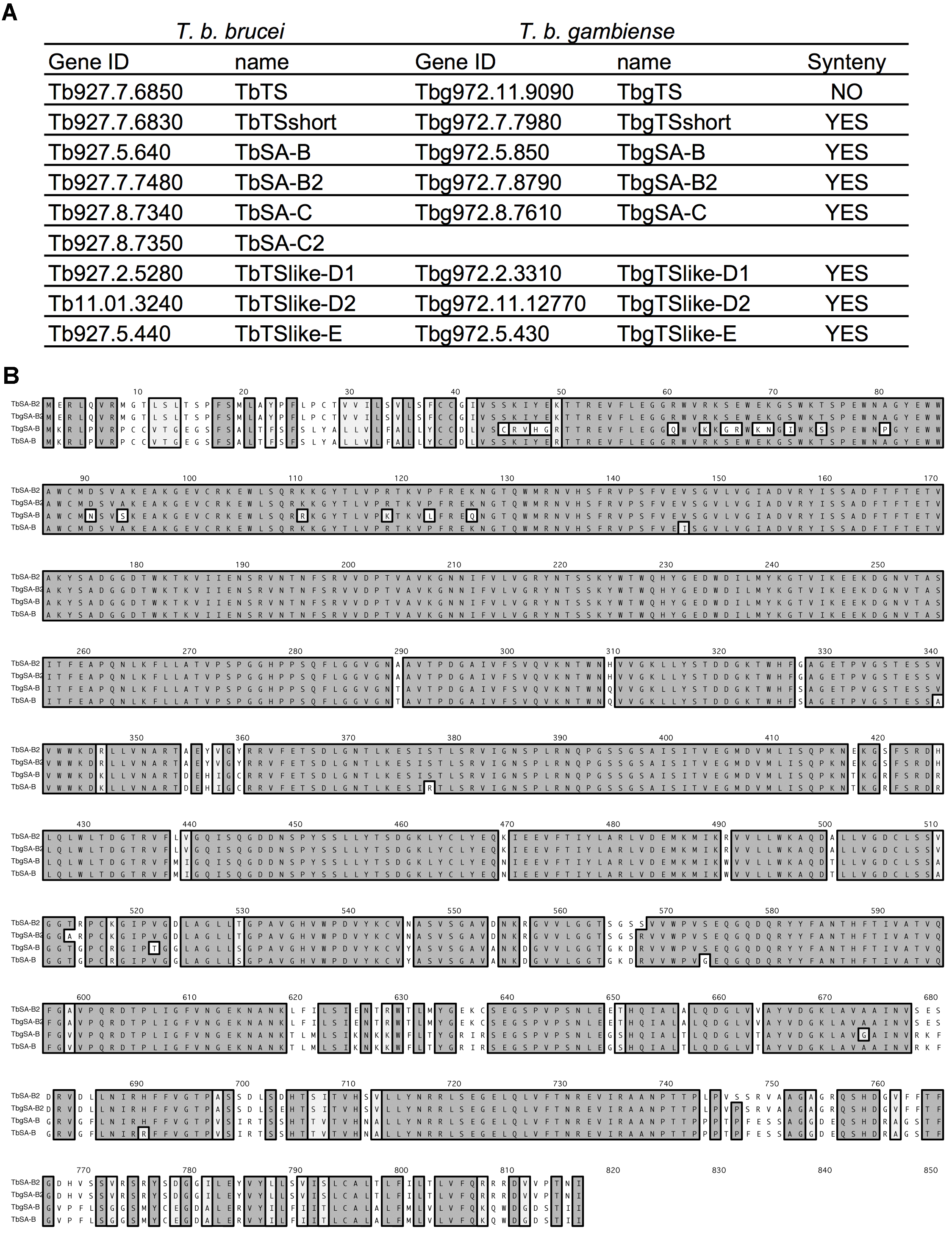

Supplement: Figure S5 — Related to Figure 7 . SA and TS genes in T. b. brucei and T. b. gambiense genomes. (A) List of the T. b. brucei SA and TS genes was established from genome database (http://tritrypdb.org/tritrypdb/) and from two previous studies on these genes [37], [39]. T. b. gambiense orthologs were found in genome database (http://tritrypdb.org/tritrypdb/). (B) Sequence alignment of SA B and SA B2 of T. b. gambiense and T. b. brucei. (TIF) [file ppat.1003710.s005.tif]

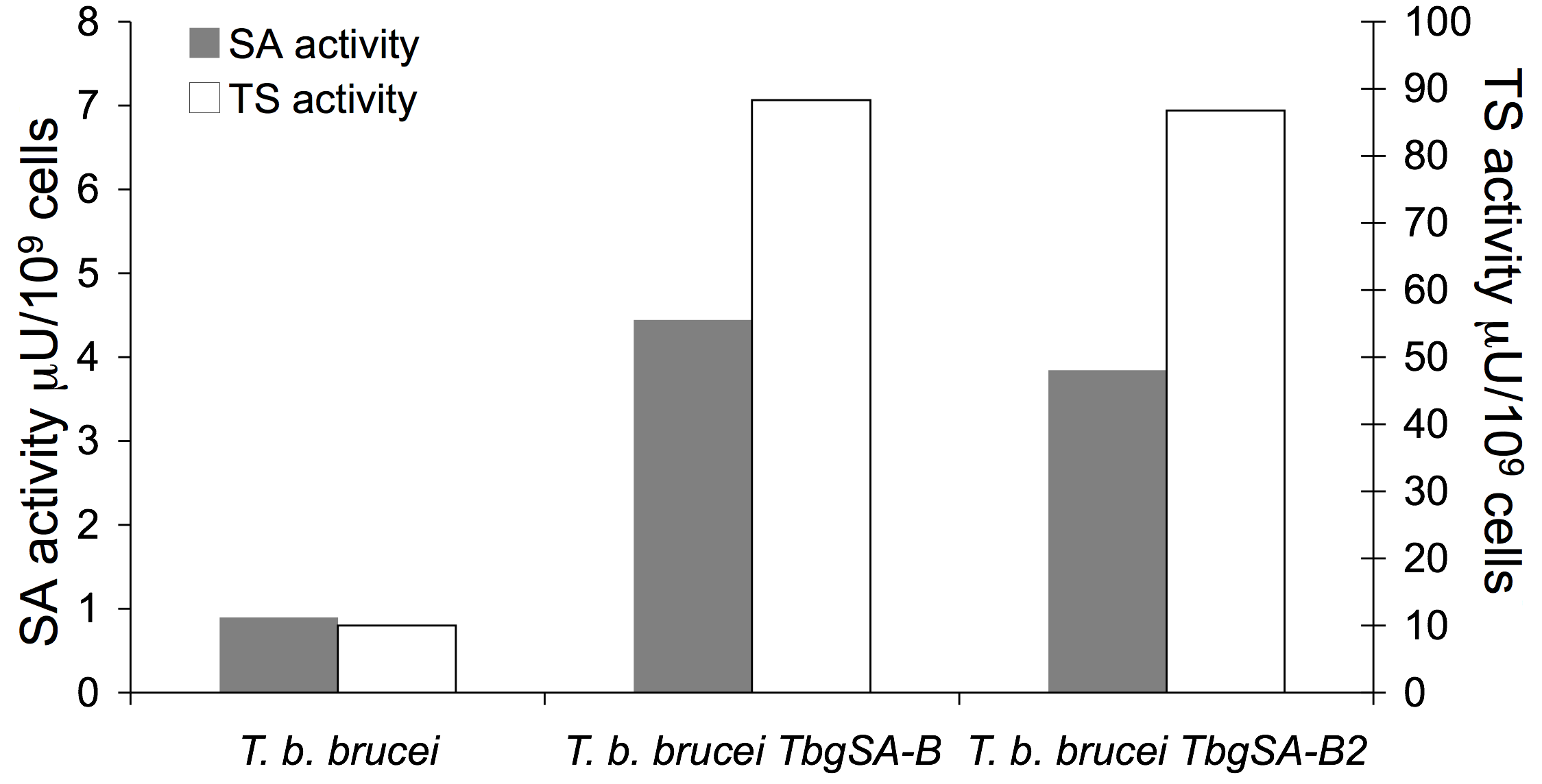

Supplement: Figure S6 — Related to Figure 7 . Overexpression of heterologous TbgSA B and TbgSA B2 in T. b. brucei BSF. SA and TS activities were measured in crude extracts of T. b. brucei TbgSA B and T. b. brucei TbgSA B2 cell lines and compared to the non-transfected cell line T. b. brucei 427 BSF. (TIF) [file ppat.1003710.s006.tif]
