# Supplementary material for: Identification of Trans-Sialidases as a Common Mediator of Endothelial Cell Activation by African Trypanosomes
Source: PLoS Pathog. 2013 Oct 10;9(10):e1003710. doi: 10.1371/journal.ppat.1003710 (PMC3795030; doi:10.1371/journal.ppat.1003710)
Supplement: Table S1 — Related to Figure 2 . Heterogeneity of endothelial cell activation by African trypanosomes and recombinant TS. a M lung, M BM, M spleen, M brain, M thymus. b HUVEC, H lung, H brain, H skin, H intestine, H appendix. (DOC) [file ppat.1003710.s007.doc]

|  | **BAE** | **Murine EC**^a^ | **Human EC**^b^ | **H PLN** | **M PLN** |
| --- | --- | --- | --- | --- | --- |
| T. b. brucei | - | - | - | - | - |
| T. vivax  T. b. gambiense | + | + | + | + | + |
| T. congolense | + | + | - | + | + |
| TS | + | - | - | + | + |
