# Supplementary material for: Identification of Trans-Sialidases as a Common Mediator of Endothelial Cell Activation by African Trypanosomes
Source: PLoS Pathog. 2013 Oct 10;9(10):e1003710. doi: 10.1371/journal.ppat.1003710 (PMC3795030; doi:10.1371/journal.ppat.1003710)
Supplement: Table S2 — Related to Figure 7 . Identification of SA or TS expressed in T. b. brucei and T. b. gambiense BSF by mass spectrometry. (A) Sialidase peptides in membrane preparations of T. b. gambiense 1135 and LiTat BSF. TbgTS-LikeD1, TbgSA-B and TbgSA-B2 are encoded by Tbg972.2.3310, Tbg972.5.850 and Tbg.7.8790 respectively. (B) Sialidase peptides in membrane preparations of T. b. brucei 427 BSF. (DOC) [file ppat.1003710.s008.doc]

**A**

| **Peptides sequence** | **TbgTS-LikeD1** | **TbgSA-B** | **TbgSA-B2** |
| --- | --- | --- | --- |
| WPVGVQGQNQR | + |  |  |
| VFESDYMGDVWTESLGTISR | + |  |  |
| IWFSDGSR | + |  |  |
| SYLLYSNNR | + |  |  |
| VSMFFGGGGNTIVMTK | + |  |  |
| SLVGELENAR | + |  |  |
| VFETSDLGNTLK |  | + | + |
| LLYSTDDGK |  | + | + |
| VPSFVEVSGVLVGIADVR |  | + | + |
| YISSADFTFTETVAK |  | + | + |
| GNNIFVLVGR |  | + | + |
| VVWPVSEQGQDQR |  | + | + |
| GVVLGGTSGSR |  |  | + |
| VFLVGQISQGDDNSPYSSLLYTSDGK |  |  | + |
| LFILSIENTR |  |  | + |
| GVVLGGTSGSR |  |  | + |
| AANPTTPLPVPSR |  |  | + |

**B**

| **Peptides sequence** | **TbTS-LikeD1** | **TbSA-B** | **TbSA-B2** |
| --- | --- | --- | --- |
| SLVGELENAR | + |  |  |
| VFESDYMGDVWTESLGTISR | + |  |  |
| IWFSDGSR | + |  |  |
| SYLLYSNNR | + |  |  |
| YISSADFTFTETVAK |  | + | + |
| VFETSDLGNTLK |  | + | + |
| GNNIFVLVGR |  | + | + |
| VIGNSPLR |  | + | + |
| VPSFVEVSGVLVGIADVR |  |  | + |
| TWHFGAGETPVGSTESSVVWWK |  |  | + |
| VVWPVSEQGQDQR |  |  | + |
| ESISTLSR |  |  | + |
| VDLLNIR |  |  | + |
| WVVLLWK |  | + |  |
| TWHFSAGETPVGSTESSVVWWK |  | + |  |
| GIPTGGLAGLLSGPAVGHVWPDVYK |  | + |  |
| AANPTTPPPTPFESSAGGDEQSHDR |  | + |  |
| VGFLNIR |  | + |  |
| WFLTYGR |  | + |  |
| LAVAAINVR |  | + |  |
