# Supplementary material for: Identification of Trans-Sialidases as a Common Mediator of Endothelial Cell Activation by African Trypanosomes
Source: PLoS Pathog. 2013 Oct 10;9(10):e1003710. doi: 10.1371/journal.ppat.1003710 (PMC3795030; doi:10.1371/journal.ppat.1003710)
Supplement: Text S1 — Supplemental experimental procedures. (DOC) [file ppat.1003710.s009.doc]

# SUPPLEMENTAL EXPERIMENTAL PROCEDURES

# Membrane preparations from *T. b. gambiense* and *T. b. brucei* BSF

10^9^ cells (*T. b. gambiense* 1135 or LiTat BSF or *T. b. brucei* 427 BSF) were osmotically lysed by incubation in 1 ml hypotonic buffer (5 mM Na_2_HPO_4_, 0.3 mM KH_2_PO_4_) containing a cocktail of protease inhibitors (Complete mini EDTA-free; Roche Diagnostics GmbH) at 4°C for 30 min and centrifuged (12000xg 10 min). The pellet was subjected to the same treatment 3 times. This insoluble material was resuspended in 100 μl of the same lysis buffer at 4°C, and 0.5 ml saline buffer (2 mM EDTA, 15.4 mM NaOH, 0.2 mM dithiothreitol) was added. After 10 min incubation, the mixture was centrifuged (12000xg 10 min). The supernatant was collected (soluble fraction) and the pellet (insoluble fraction) resuspended in 50 μl distilled water, supplemented with 50 μl SDS 2%. 50 μl of each of the 2 fractions was mixed with 15 μl Laemmli buffer 4X (200 mM Tris-HCl pH=6.8; 40% glycerol; 4% SDS; 10% β-mercaptoethanol; 0.04% bromophenol blue) and incubated at 100°C for 10 min. These two samples were analysed by SDS-PAGE.

# Protein identification by mass spectrometry

Silver-stained protein bands from 60 to 150 kDa were washed in H_2_O/ACN (50:50) until destained. The solvent mixture was removed and replaced with ACN. The gel pieces were shrunk, and ACN was removed, and they were dried in a vacuum centrifuge. Gel pieces were rehydrated in 10 ng μl-1 trypsin (Sigma–Aldrich, St Louis, MO, USA) in 50 mm NH_4_HCO_3_ and incubated at 37°C overnight. The supernatant was removed and stored at −20°C, and the gel pieces were incubated in 50 mm NH_4_HCO_3_ at room temperature for 15 min. The second supernatant was pooled with the previous one, and H_2_O/ACN/HCOOH (47.5:47.5:5) solution was added to the gel pieces for 15 min. This step was repeated twice. Supernatants were pooled and concentrated in a vacuum centrifuge to a final volume of 25 μl. Digests were finally acidified by adding 1.2 μl acetic acid and stored at −20°C.

Peptide mixtures were analysed by online nano-HPLC (LC Packings, Amsterdam, Netherlands) coupled to a nanospray LCQ Deca XP Plus ion trap mass spectrometer (Thermo-Finnigan, San Jose, CA, USA). Peptides were separated on a 75 μm id × 15 cm C18 PepMapTM column (LC Packings, Amsterdam, Netherlands). The flow rate was set at 200 nl/min. Peptides were eluted using a 5–40% linear gradient of solvent B in 35 min (solvent A was 0.1% formic acid in 5% ACN, and solvent B was 0.1% formic acid in 80% ACN). The mass spectrometer was operated in positive ion mode at a 2 kV needle voltage and 3 V capillary voltage. Data was acquired in a single run in data-dependent mode, alternating an MS scan over the range *m*/*z* 150–2000, a zoom scan, and an MS/MS scan of the most intense ion in the survey scan. MS/MS data were acquired using a two-*m*/*z*-unit ion isolation window and 35% relative collision energy.

Peptides were identified by a BLAST search by SEQUEST through the Bioworks 3.3.1 interface (ThermoFinnigan) against the GeneDB_Tcongolense_Protein_v1.fasta database (from the Wellcome Sanger Center) and a local Sialidases_PRT.fasta database containing the IL3000 sialidase sequences. Methionine oxidation (+16 Da) and cysteine carbamidomethylation (+57 Da) were considered differential modifications. Two missed trypsin cleavages were allowed. Only peptides with Xcorr higher than 1.9 (single charge), 2.2 (double charge), and 3.75 (triple charge) were retained. In all cases, DeltaCn had to be above 0.1 and peptide *p*-value below 1.10^-3^. Proteins identified by a single peptide were rejected. Peptide redundancy was eliminated so as to list the minimum number of proteins covering all detected peptides.

# Enzyme activity assays

Sialidase activity was determined by measuring the fluorescence of 4-methylumbiliferone released by the hydrolysis of 2’-(4-methylumbelliferyl)-α-D-*N*-acetylneuraminic acid (MUNeu5Ac, Sigma). Assays were performed in 50 mM Bis-Tris buffer, pH 7.0, with 0.2mM mM MUNeu5Ac in a final volume of 100 μl in black 96-well-plates (Nunc). Activity was monitored every 45 sec after substrate addition for 2 h by measuring MU release (λex 365 nm, λem 430 nm) with an Optima microplate reader (BMG Labtech, Germany) calibrated with MU standard solutions. One unit (U) of sialidase activity equals 1 μmol MU released per min, which is equivalent to 1 μmol sialic acid released per min.

Trans-sialidase activity was monitored by incubating a 25 μl enzyme sample in 50 mM Bis-Tris buffer, pH 7.0, containing 1 mM α2,3-sialyllactose (α2,3-SL, Dextra Laboratories) as the donor and 0.5 mM MU-β-D-galactopyranoside (MUGal, Sigma) as the acceptor in a final volume of 50 μl at 37 °C for 2 h. Adding 1 ml ice-cold water terminated the reaction. The mixture was incubated with 300 μl Q-Sepharose Fast Flow resin. After washing, the sialylated product was eluted with 700 μl 1 M HCl, hydrolyzed at 95°C for 45 min, and cooled on ice. The samples were neutralized, adjusted to pH 10, and MU release was measured by endpoint as stated above. One unit of trans-sialidase activity equals 1 μmol MU released per minute, equivalent to 1 μmol sialic acid transferred per minute.

Activity was measured on parasite lysates (expressed as μU per 10^9^ cells lysed) or purified recombinant proteins (expressed as mU/mg recombinant protein). For all assays, product formation was linear with respect to time and protein amount; controls were measured in the absence of enzyme.
